# Mapping out the Aqueous Surface Chemistry of Metal Oxide Nanocrystals: Carboxylate, Phosphonate, and Catecholate Ligands

**DOI:** 10.1021/jacsau.1c00565

**Published:** 2022-03-04

**Authors:** Loren Deblock, Eline Goossens, Rohan Pokratath, Klaartje De Buysser, Jonathan De Roo

**Affiliations:** † Department of Chemistry, Ghent University, 9000 Ghent, Belgium; ‡ Department of Chemistry, University of Basel, 4058 Basel, Switzerland

**Keywords:** nanoparticle, catechol, carboxylic acid, phosphonic acid, hafnium oxide

## Abstract

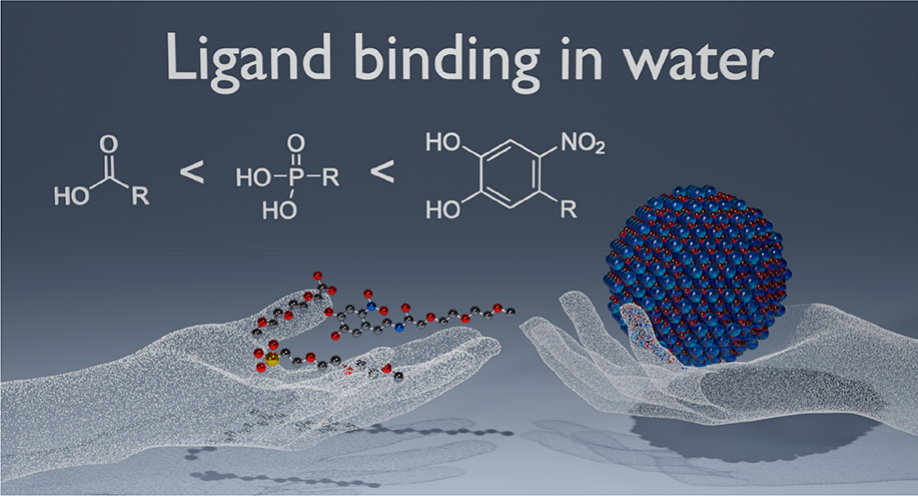

Iron oxide and hafnium oxide nanocrystals are two of the few successful examples of inorganic nanocrystals used in a clinical setting. Although crucial to their application, their aqueous surface chemistry is not fully understood. The literature contains conflicting reports regarding the optimum binding group. To alleviate these inconsistencies, we set out to systematically investigate the interaction of carboxylic acids, phosphonic acids, and catechols to metal oxide nanocrystals in polar media. Using nuclear magnetic resonance spectroscopy and dynamic light scattering, we map out the pH-dependent binding affinity of the ligands toward hafnium oxide nanocrystals (an NMR-compatible model system). Carboxylic acids easily desorb in water from the surface and only provide limited colloidal stability from pH 2 to pH 6. Phosphonic acids, on the other hand, provide colloidal stability over a broader pH range but also feature a pH-dependent desorption from the surface. They are most suited for acidic to neutral environments (pH <8). Finally, nitrocatechol derivatives provide a tightly bound ligand shell and colloidal stability at physiological and basic pH (6–10). Whereas dynamically bound ligands (carboxylates and phosphonates) do not provide colloidal stability in phosphate-buffered saline, the tightly bound nitrocatechols provide long-term stability. We thus shed light on the complex ligand binding dynamics on metal oxide nanocrystals in aqueous environments. Finally, we provide a practical colloidal stability map, guiding researchers to rationally design ligands for their desired application.

## Introduction

Colloidal nanocrystals (NCs) have been considered for a multitude of biomedical applications, such as bioimaging,^1−3^ drug delivery,^4,5^ photothermal therapy,^6^ and radiotherapy enhancement.^7,8^ These NCs are typically hybrid objects, consisting of an inorganic core capped with organic ligands. Inorganic ligands are more useful for devices.^9^ Ligands determine the interaction between NC and solvent and the stability of the nanocolloid.^10^ In the case of biomedical applications, controlling the NC surface chemistry is key as it will play a role in particle agglomeration, cellular uptake,^11−13^ protein repelling or adsorption,^12,14,15^ cytotoxicity,^12,16^ circulation time,^15,17^ and targeted approaches.^18−20^ Although surface chemistry is important for all types of NCs (chalcogenides, pnictides, halides, and metal NCs), not one solution fits all. For example, thiolates and thiols have a strong binding affinity to Au and CdSe NCs but interact poorly with metal oxide NCs.^21^

Metal oxide NCs have been particularly successful in nanomedicine. Three types of inorganic NCs have achieved clinical translation, and two of them are oxides: iron oxide and hafnium oxide.^5^ These particles are often first synthesized in nonpolar solvents and stabilized by surfactants (usually with a carboxylate or phosphonate headgroup and an aliphatic tail). Carboxylic acids (e.g., oleic acid) dissociate on the metal oxide surface, with carboxylates binding to surface metal sites and protons to surface oxygen atoms.^22−24^ This binding motif is written as NC(XX′) as both proton and carboxylate are X-type ligands.^25^ In nonpolar solvents, carboxylic acids are quantitatively exchanged by phosphonic acids in an X-for-X ligand exchange process.^26^ Indeed, phosphonic acids are very strong ligands for oxide surfaces.^27,28^ In contrast, catechol was found to be a rather weak ligand, only able to exchange a minor fraction of oleic acid.^29^ There is thus a clear order in binding strength in nonpolar solvents: catechol < carboxylic acid < phosphonic acid.

In aqueous (or other polar) environments, the order is less clear and further complicated by variable factors such as pH and salt concentration. For example, carboxylic acids are frequently used to stabilize metal oxide NCs in water.^30−34^ They are able to provide colloidal stability in static systems with no competing ligands but not in phosphate-buffered saline (PBS) or cell culture media.^34^ The binding affinity is significantly increased for multidentate carboxylate ligands such as polymers.^35,36^ In general, literature reports agree that carboxylic acids are the weakest ligands in water, weaker than phosphonic acids or catechols.^37^ The literature is, however, inconclusive as to whether phosphonic acids or catechols are the best ligand. For example, Guénin et al. concluded from Fourier transform infrared spectroscopy and density functional theory calculations that caffeic acid (a catechol) has a higher affinity for 9 nm iron oxide NCs than a bisphosphonate at physiological pH.^38^ In contrast, Zeininger et al. determined that phosphonic acids bind more strongly than catechols to TiO_2_ NCs in isopropyl alcohol at pH 7.^39^ Okada et al. came to the same conclusion on 9 nm iron oxide NCs by performing phase-transfer experiments using polar and nonpolar phosphonic acids and catechols.^40^ It is clear that, despite the common usage of phosphonic acids and catechols in metal oxide NC functionalization, there is no clear consensus on the relative binding affinity. Furthermore, a direct link between ligand binding equilibria and the final colloidal stability of the NCs is usually not made.

In this paper, we aim to unambiguously establish the binding affinity order and provide the correct surface chemistry for optimal application in nanomedicine. We chose HfO_2_ NCs as our model system for two reasons: (1) it is a relevant material in nanomedicine, and (2) it is compatible with solution nuclear magnetic resonance (NMR) spectroscopy. The latter has proven to be a very powerful tool to study nanocrystal surface chemistry,^41−45^ providing complementary information to solid-state NMR spectroscopy.^44,47^ Unfortunately, iron oxide NCs interfere with magnetic fields and cannot be studied in NMR. HfO_2_ NCs are thus an ideal starting point also because their surface chemistry has already been extensively studied in nonpolar solvents using NMR spectroscopy.^22,23^ First, we evaluate the ligand exchange of the native carboxylic acid ligands for phosphonic acid and catechol ligands, using ^1^H and ^31^P NMR spectroscopy. Importantly, we use the same polyethylene glycol ligand chain for all three binding groups, thus ensuring that we can directly compare the binding groups to each other. Next, we assess the influence of solvent (methanol vs water) and the pH on ligand binding. Furthermore, we used NMR and dynamic light scattering (DLS) to determine the colloidal stability provided by the different ligand types in aqueous and buffer environments and directly correlate this to ligand binding dynamics. Finally, we constructed a colloidal stability map, showing which binding group provides colloidal stability as a function of pH. This practical guide will help researchers in designing future surface chemistries.

## Results

### HfO_2_-MEEAA Model System

**Figure 1:**
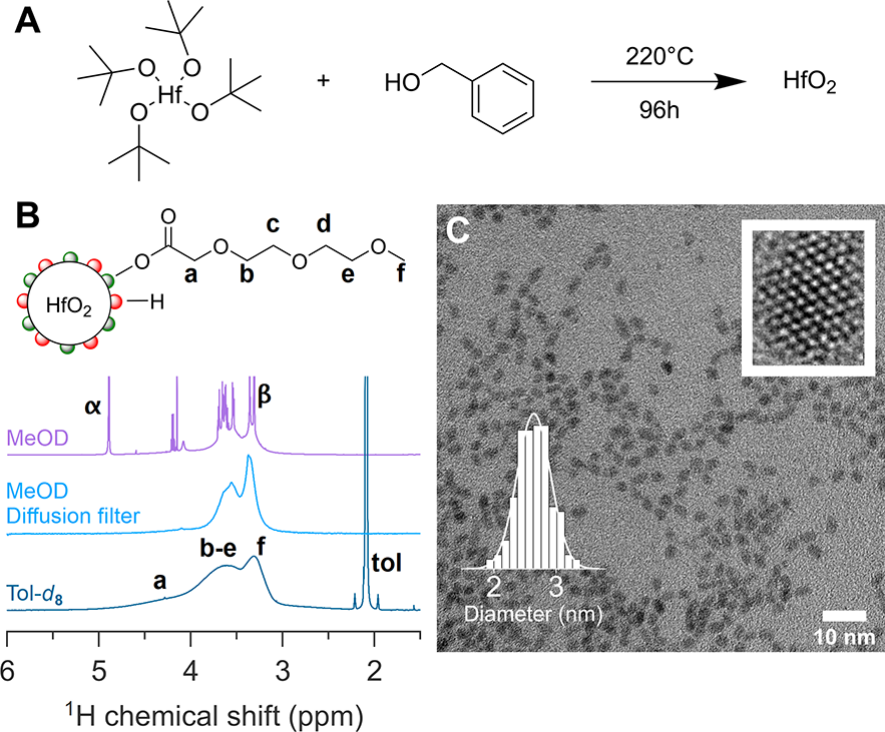
(A) Solvothermal synthesis of HfO_2_ nanocrystals starting from 1 equiv of Hf(O-*t*Bu)_4_ and 80 equiv of benzyl alcohol. (B) ^1^H NMR spectra (normal or diffusion filtered) of MEEAA-functionalized HfO_2_ NCs in different solvents. The α and β resonances belong to the residual hydroxyl and methyl groups of methanol, respectively. (C) TEM image of the synthesized HfO_2_ NCs. The NC diameter of the quasi-spherical NCs was calculated after measuring the surface area of at least 150 NCs and calculated the diameter as if it were a circle. A size distribution histogram and a zoomed-in image of a singular NC can be seen, respectively, in the bottom left and the top right corner.

We synthesized HfO_2_ nanocrystals (NCs) from hafnium *tert*-butoxide and benzyl alcohol at 220 °C via an established solvothermal process (Figure 1A).^48^ The HfO_2_ surface was functionalized with 2-[2-(2-methoxyethoxy)ethoxy]acetic acid (MEEAA; see Figure 1B) to stabilize the nanocrystals in toluene, and all unbound ligands were removed as described previously.^45^ The nanocrystals have a diameter of 2.64 ± 0.81 nm (μ ± 3σ) according to transmission electron microscopy (TEM) (Figure 1C) and possess the monoclinic (*P*2_1_/*c*) crystal structure according to X-ray total scattering and pair distribution function (PDF) analysis (Figure S1). The ^1^H NMR spectrum of the nanocrystal dispersion in toluene-*d*_8_ only shows broadened resonances, assigned to bound ligands (Figure 1B). Indeed, spectral broadening is a typical attribute of bound ligands due to both homogeneous broadening (*T*_2_ relaxation) and heterogeneous broadening (imperfect solvation of the ligand shell).^45^

The nanocrystals can also be dispersed in ethanol, methanol, and water and are thus an ideal starting point for our investigation into ligand binding behavior in polar solvents. In methanol-*d*_4_ (MeOD), the ^1^H NMR spectrum looks markedly different, with sharp signals superimposed on the broad resonances (Figure 1B). We assign the sharp signals to autodesorbed ligands, corroborated by the appearance of two sets of resonances in diffusion ordered spectroscopy (DOSY, Figure S2). DOSY allows one to separate (overlapping) NMR resonances according to their diffusion coefficient and thus separates the free ligands (quickly diffusing) from ligands bound to NCs (slowly diffusing).^49^ In Figure S2, the resonances with a diffusion coefficient *D* = 95 m^2^/s represent the bound ligands, and the resonances with *D* = 450 m^2^/s belong to the free ligands. We can selectively observe the bound ligands in a diffusion-filtered spectrum (Figure 1B), and upon close inspection, one observes that the bound MEEAA resonances in methanol-*d*_4_ are slightly sharper than the ones in toluene-*d*_8_, indicating a better solvation of the ligand shell in methanol.^45^ Given that all ligands were bound in toluene, we infer that the solvent clearly plays a role in the adsorption–desorption equilibrium, for instance, by changing the solubility of the ligand.^50^ Indeed, even a higher fraction of MEEAA ligands desorbs in water, D_2_O (see Figure S3), but the nanocrystals remain visibly stable in the pH range of 2–6. The NCs quickly precipitate at pH >6, and therefore, MEEAA is not a suitable ligand for many biomedical applications, which typically require stability at physiological conditions (pH 7.4).

Note that, aside from the main MEEAA signals, we also observed broad resonances with low intensity in the aromatic region of the ^1^H spectrum (Figure S4), assigned to benzoate ligands. Benzoic acid was previously identified as a side product of the nanocrystal synthesis and was found to be adsorbed on the nanocrystal surface.^48^ Functionalization of the surface with MEEAA after synthesis clearly did not remove all benzoate from the surface, and a small fraction remains present.

### Competitive Binding of Phosphonic Acids

**Figure 2:**
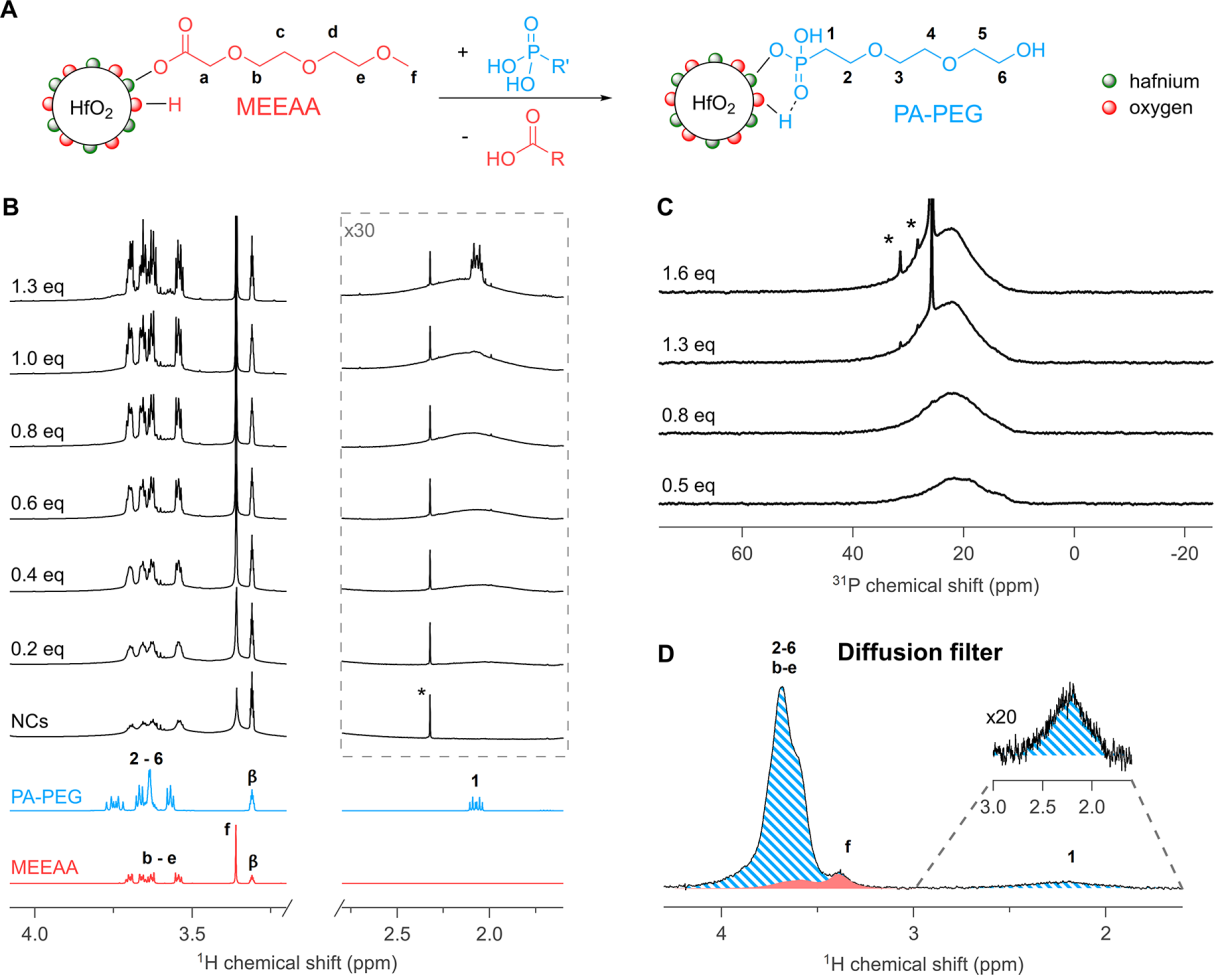
(A) Ligand exchange performed between MEEAA-functionalized NCs and PA-PEG. (B) ^1^H NMR reference spectra in MeOD of the free ligands as reference and the stepwise titration of MEEAA-functionalized NCs with PA-PEG, equivalents are with respect to the total amount of MEEAA present. (C) ^31^P NMR spectra (4096 scans) in MeOD for the stepwise titration of MEEAA-functionalized NCs with PA-PEG; broadened signals are indicative of NC binding. (D) Diffusion-filtered ^1^H NMR spectra of MEEAA-functionalized NCs in MeOD after addition of 1.3 equiv of PA-PEG. Signals arising from bound MEEAA are denoted in red, and signals arising from PA-PEG are denoted in striped blue. *C*_NC_ = 1210 μmol L^–1^, corresponding to 34 mg NCs of this size in 0.5 mL of MeOD. Resonances denoted as * are unidentified impurities.

To evaluate the binding strength of phosphonic acids in methanol, we chose (2-(2-(2-hydroxyethoxy)ethoxy)ethyl)phosphonic acid (PA-PEG) as a ligand with structure comparable to that of MEEAA (Figure 2A). In the ^1^H NMR spectrum, resonance **1** of PA-PEG has a chemical shift of 2.05 ppm, clearly separated from the MEEAA resonances, allowing us to selectively monitor the binding of PA-PEG (Figure 2B). As MEEAA has a methoxy group and PA-PEG does not, resonance **f** is used to gain selective information about the binding of MEEAA. The other resonances (**2**–**6** and **b**–**e**) overlap.

Starting from MEEAA-stabilized HfO_2_ nanocrystals in methanol-*d*_4_, we add PA-PEG in steps while monitoring the ^1^H NMR spectrum; see Figure 2B (full range spectra and additional titration points in Figure S5). During the titration, we observe the gradual appearance of a broad resonance around 2.2 ppm, assigned to resonance **1** of bound PA-PEG. Concomitantly, resonance **f** becomes more narrow (Figure 2B), indicating the removal of MEEAA from the nanocrystal surface. Within the aromatic region, we also observe desorption of benzoic acid (Figure S5). We conclude that PA-PEG is effectively displacing MEEAA and benzoic acid. Given the absence of free phosphonic acid, the exchange is quantitative for most of the titration. After addition of 1.3 equiv of phosphonic acid, sharp signals appear in the same region, indicating that there is now free PA-PEG present. The same conclusions are drawn from the ^31^P spectra, as well (Figure 2C), where first a broad signal grows in intensity and a sharp ^31^P signal appears after adding more than 1 equiv. Interestingly, the bound PA-PEG resonance still increases slightly in intensity between 1.3 and 1.6 equiv. We infer that the last part of the exchange does not proceed quantitatively, and the remaining carboxylate ligands are more difficult to remove. This observation implies that the binding affinity (Δ*G*_ads_) is not a single, fixed quantity for all MEEAA ligands but rather a distribution, as has been previously shown for CdSe nanocrystals.^51^

Unfortunately, the NMR spectra feature a lot of spectral overlap, with superimposed signals of free and bound ligands. At the end of the titration, the resonances of free ligands dominate the spectrum. To gain more insight into the composition of the ligand shell at that point, we turn to the diffusion-filtered spectrum (Figure 2D, full range in Figure S6). Whereas resonances **2**–**6** overlap with resonances **b**–**e**, the clear presence of resonance **f** indicates the presence of residual MEEAA on the surface. Based on the diffusion-filtered profile of bound MEEAA (Figure S4), we assigned the part of the spectrum belonging to MEEAA as the red shaded area. The blue striped area is assigned to PA-PEG. There is also still benzoate present on the surface according to the regular ^1^H spectrum, but the signal-to-noise of these resonances was too low in the diffusion-filtered spectrum due to their faster *T*_2_ relaxation (because of rigidity and proximity to the surface). The above results thus confirm that the exchange between the carboxylate ligands and PA-PEG does not go to completion in methanol. This conclusion stands in contrast to the relative binding affinities of fatty acids and alkylphosphonic acids in nonpolar solvents, where phosphonic acids quantitatively replace carboxylic acids in a 1:1 stoichiometry.^26,52,53^

**Figure 3:**
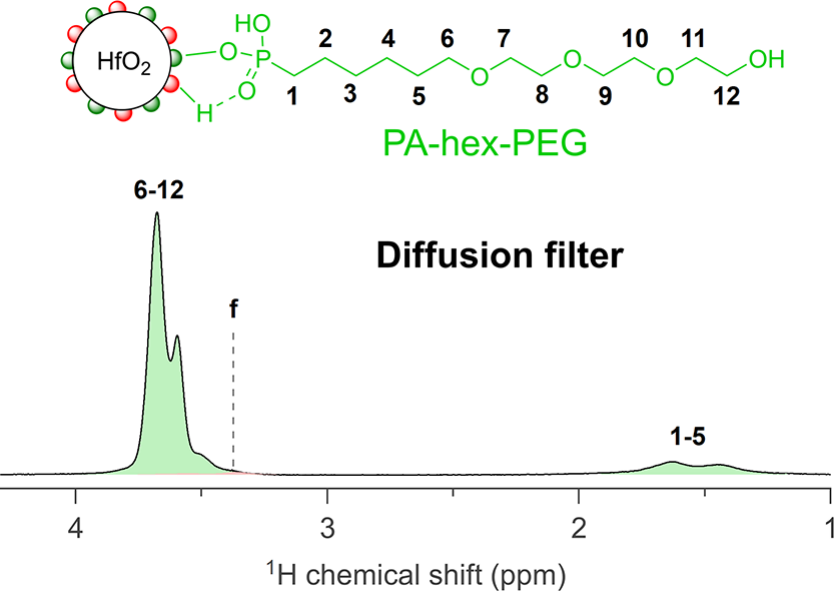
Diffusion-filtered ^1^H NMR spectrum of the NC suspension in MeOD with 1.3 equiv of PA-hex-PEG added.

The autodesorption of MEEAA in methanol already indicated that the ligand solubility can change the binding affinity. Indeed, ligand binding is an equilibrium process, governed by the chemical potential of each species:
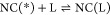
1Therefore, this adsorption–desorption equilibrium is dependent on the chemical potential of the free ligand (and thus the solubility).^50^ To further explore this concept in practice, we designed a ligand shell architecture that would mimic a micelle, having both a hydrophobic and a hydrophilic segment. To this end, we chose the ligand (6-[2-[2-(2-hydroxyethoxy)ethoxy]ethoxy]hexyl)phosphonic acid (PA-hex-PEG); see Figure 3. We hypothesized that this ligand would more readily self-assemble on the nanocrystal surface as the hydrophobic region decreases the solubility in polar solvents. Using PA-hex-PEG, we performed the same titration experiment as before. The results are generally quite similar, showing the exchange of carboxylate ligands for PA-hex-PEG (Figure S7 and Figure S8). However, this time, most benzoate ligands are removed by PA-hex-PEG (although not completely) and hardly any MEEAA resonances are detectable in the diffusion-filtered spectrum (Figure 3). We conclude that, indeed, the competitive binding can be manipulated by changing the ligand solubility. Interestingly, benzoic acid appears to have a binding affinity higher than that of MEEAA, which could be ascribed to both its higher acidity and its lower solubility in methanol.

### Purification and Transfer to Water of Phosphonate-Capped Nanocrystals

**Figure 4:**
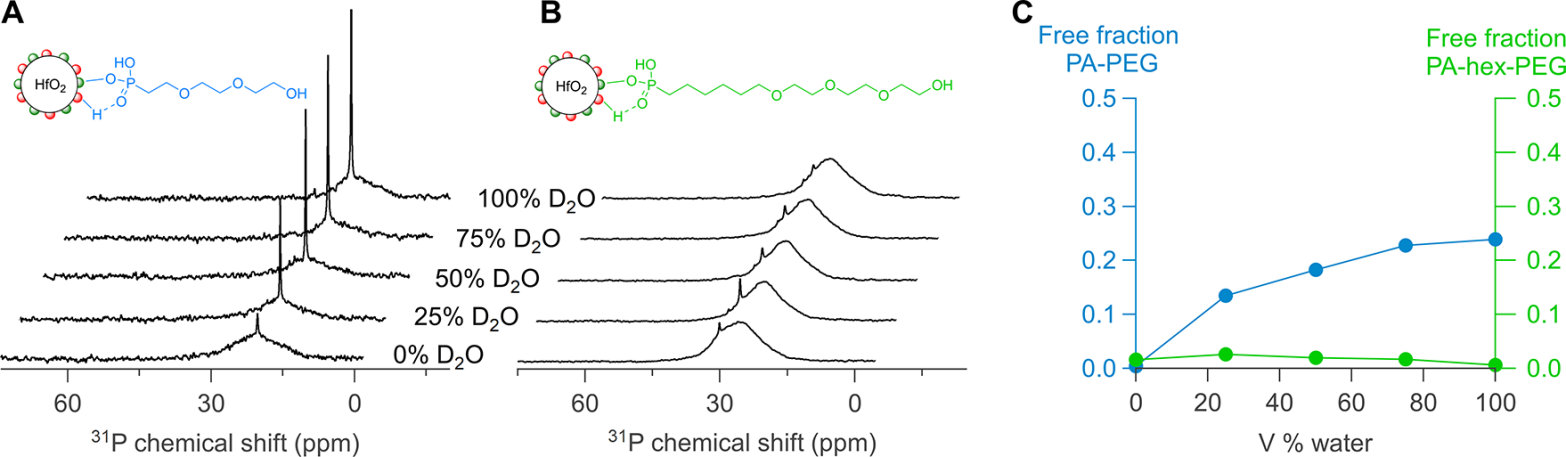
(A,B) ^31^P NMR spectra of PA-PEG- and PA-hex-PEG-functionalized NCs at different D_2_O volume %. (C) Free ligand fraction for PA-PEG and PA-hex-PEG at different D_2_O volume %, determined by peak deconvolution.

With our final goal in mind of providing a stable surface chemistry for biomedical applications, we sought to purify our dispersions and disperse them in aqueous media. Precipitation–redispersion cycles are the most common way of purifying nanocrystals. However, the great versatility of the ethylene glycol segment provides colloidal stability in a broad range of solvents, and nonsolvents, such as hexane, do not mix well with methanol. Therefore, we choose to purify our dispersion using spin filtration, a form of ultrafiltration. The technique is based on semipermeable membranes (like dialysis), where small molecules can pass through the pores but large nanocrystals cannot. By performing the separation in a centrifuge, purification is expedited. First, the nanocrystal suspension is placed in the spin filter and further diluted with pure methanol. Figure S9 shows that dilution does not induce ligand desorption. After filtration, a concentrated dispersion of nanocrystals is retrieved. The purification is successful, as shown by the removal of almost all unbound species after three purification cycles (Figure S10). A comparison of the diffusion-filtered spectra before and after spin filtration shows a perfect match, proving that the purification did not change the ligand shell composition (Figure S10). Interesting differences between PA-PEG and PA-hex-PEG were observed when the solvent composition gradually changed from pure methanol-*d*_4_ to D_2_O (Figure 4). Whereas PA-PEG gradually desorbs from the surface when increasing the water content, PA-hex-PEG remains tightly bound. This further underscores our hypothesis that PA-hex-PEG behaves as a micelle mimic, avoiding contact of the hydrophobic segment with water. By binding the nanocrystal surface, the PA-hex-PEG ligand creates a hydrophobic inner shell with alkyl–alkyl interactions and a hydrophilic outer shell with ethylene glycol moieties as the hydrogen bond acceptor for water molecules. PA-PEG, on the other hand, is highly water-soluble, and thus the affinity for the hafnium oxide surface decreases upon increasing the water content.

### Synthesis and Binding of Catechols

**Scheme 1:**
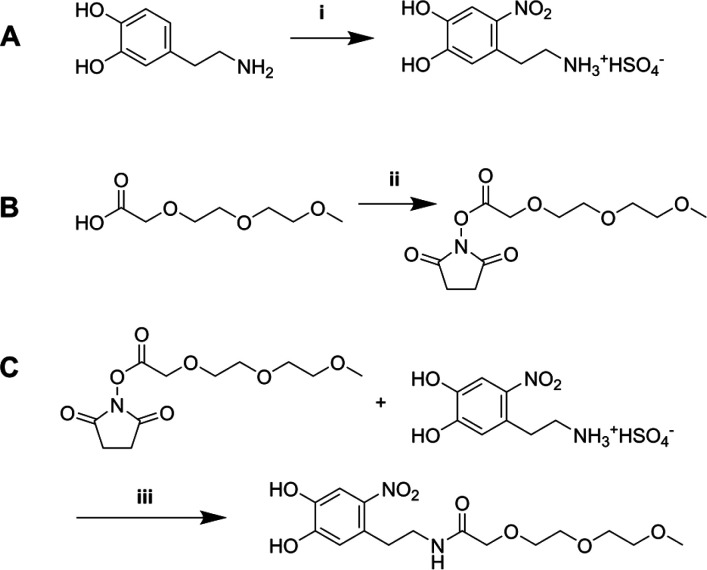
(A) Synthesis of Nitrodopamine Hemisulfate, (B) Synthesis of MEEAA-NHS, and (C) Synthesis of Nitrodopamine-mPEG, Reagents and conditions: (i) NaNO_2_, 20% H_2_SO_4_, H_2_O, 0 °C to RT, 12 h, 50%; (ii) NHS, DCC, DMAP, dry THF, 0 °C to RT, 12 h, 83%; (iii) NMM, dry DMF, RT, 48 h, 75%.

We synthesized *N*-(4,5-dihydroxy-2-nitrophenethyl)-2-(2-(2-methoxyethoxy)ethoxy)acetamide (nitrodopamine-mPEG); see Scheme 1. We opted for a nitrocatechol instead of an unsubstituted catechol because the nitro group decreases the catechol p*K*_a_ values and improves the oxidative stability of the catechol. Nitrodopamine was also shown to provide colloidal stability to iron oxide nanoparticles better than unsubstituted dopamine.^54^ Starting from dopamine hydrochloride, a one-step nitration reaction results in the formation of nitrodopamine hemisulfate. Separately, MEEAA was converted in an activated *N*-hydroxysuccinimide ester (MEEAA-NHS) using *N*,*N*′-dicyclohexylcarbodiimide (DCC), *N*-hydroxysuccinimide (NHS), and 4-dimethylaminopyridine (DMAP). The coupling between nitrodopamine hemisulfate and MEEAA-NHS was performed using *N*-methylmorpholine (NMM) acting as a non-nucleophilic base. The final nitrodopamine-mPEG ligand was purified using preparative high-performance liquid chromatography (HPLC) and fully characterized with electrospray ionization/high-resolution mass spectrometry (ESI-HRMS) and NMR spectroscopy (see Supporting Information).

When we performed a similar competitive binding experiment as before (addition of nitrodopamine-mPEG to MEEAA-capped HfO_2_ nanocrystals in methanol-*d*_4_), we found that 1 equiv of nitrodopamine-mPEG was unable to effectively compete for the nanocrystal surface; even at merely 0.4 equiv of catechol, freely diffusing nitrodopamine-mPEG signals could be observed (Figure S11). Such a low binding affinity was highly unexpected, given many reports of successful surface functionalization with catechols.^40,54,57−61^ However, in these reports, water or biological buffers were used as the solvent. Therefore, we designed a competitive binding experiment directly in water. Fortunately, MEEAA-stabilized HfO_2_ nanocrystals are stable in D_2_O, even though a significant portion of MEEAA is desorbed (Figure S3). The addition of 1 equiv nitrodopamine-mPEG, without adjusting the pH, results in a turbid suspension with pH at 2. When the pH is adjusted to pH 5, a stable suspension is obtained. To allow for a systematic, gradual addition of nitrodopamine-mPEG, we prepared a stock solution of nitrodopamine-mPEG with 2 equiv of NaOD to doubly deprotonate the catechol. Addition of the base changes the color of the solution from light yellow to a deep burgundy (Figure S12). We added this stock solution to a suspension of MEEAA-stabilized HfO_2_ in steps of 0.5 equiv and recorded both standard ^1^H NMR and diffusion-filtered NMR spectra. After addition of 0.5 equiv, we did not observe any sharp signals belonging to nitrodopamine-mPEG, whereas we did observe desorbed benzoic acid and desorbed MEEAA (Figure S13). In the diffusion-filtered spectrum, we also see a clear change. In the aromatic region, the broad signals of benzoate are replaced by the broad signals of nitrodopamine-mPEG. The peak shape of the region at 3–4 ppm is also altered (Figure S14). The exchange continues as more nitrodopamine-mPEG is added, and upon addition of 1.5 equiv, sharp (unbound) nitrodopamine-mPEG signals are detected (Figure S13). The suspension remains stable despite the pH being 10.3 at this point in the titration, providing further evidence that the ligand exchange was successful as MEEAA-stabilized nanocrystals would precipitate at pH >6. We conclude that nitrodopamine-mPEG can quantitatively displace MEEAA from the nanocrystal surface if the pH is >5.

**Figure 5:**
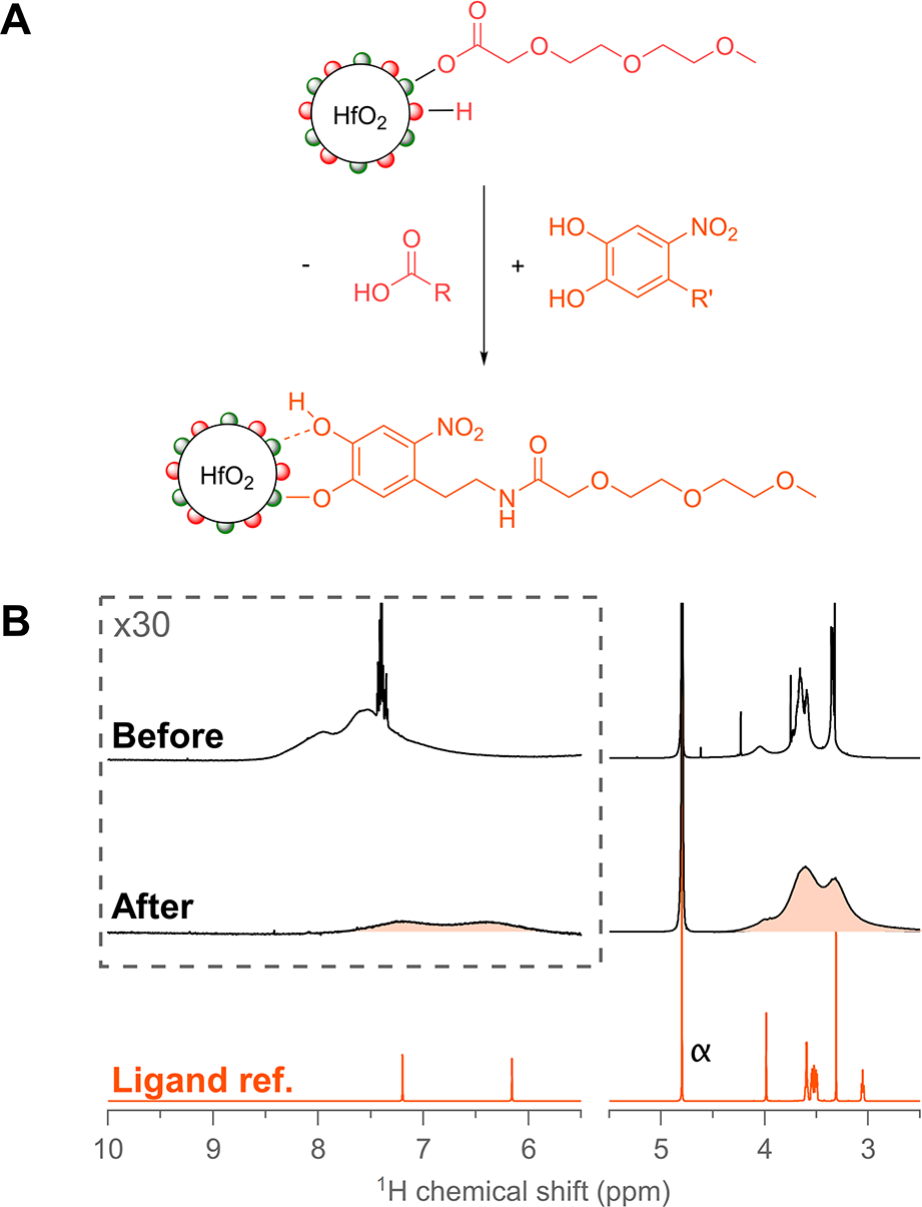
(A) Ligand exchange performed between MEEAA-functionalized NCs and nitrodopamine-mPEG. (B) ^1^H NMR spectra before and after the ligand exchange titration performed in D_2_O with nitrodopamine-mPEG. First, 1.5 equiv of nitrodopamine-mPEG was added, and the pH was kept above 5 at all times during addition. The purified nitrodopamine-functionalized NC spectrum was measured at pH 7.4. The reference spectrum of nitrodopamine-mPEG is also shown. *C*_NC_ = 128 μmol L^–1^, corresponding to 14.4 mg NCs of this size in 2 mL of D_2_O.

The NCs were again purified using multiple cycles of spin filtration, using Milli-Q water as solvent, until the filtrate was nearly colorless (Figure S15). The concentrate was evaporated and redispersed in D_2_O, and the pH was adjusted to 7.4. Figure 5B contains a regular ^1^H NMR spectrum of the purified suspension; the purity of the sample is striking, and only broadened resonances pertaining to nitrodopamine-mPEG are observed. Based on a thermogravimetric analysis of mass loss of 23%, we calculated a nitrodopamine-mPEG ligand density of 2.14 nm^–2^ at the nanocrystal surface. We conclude that nitrodopamine-mPEG forms a tightly bound ligand shell on the nanocrystals at physiological pH with no signs of desorption.

### pH Dependence of Ligand Binding

**Figure 6:**
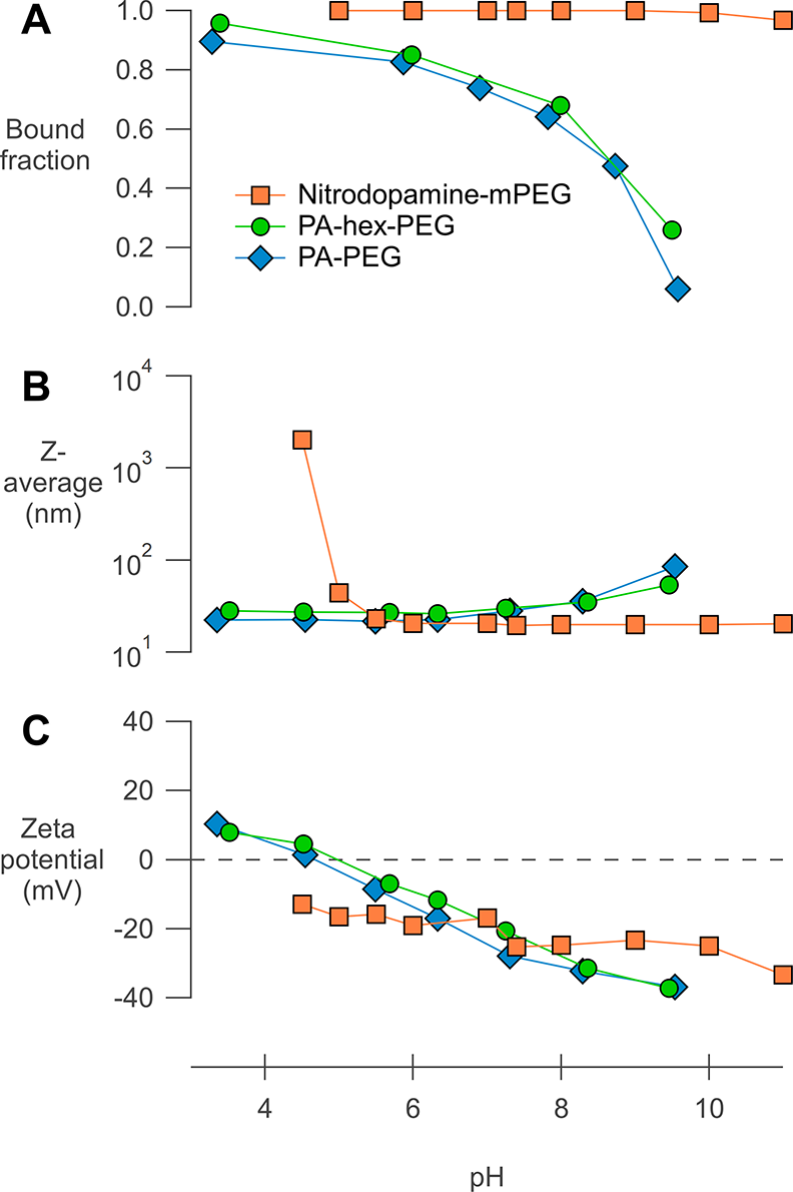
Effect of pH on ligand binding and stability in water for purified NCs functionalized with PA-PEG, PA-hex-PEG, and nitrodopamine-mPEG. (A) Bound ligand fraction in D_2_O based on NMR peak deconvolution at different pH values. (B) *Z*-average value of NCs in DLS at different pH values. (C) Zeta-potential of the NCs at different pH values. All measurements were performed at constant ionic strength (0.01 mol L^–1^ NaCl) at 25 °C.

As it is obvious that pH plays a crucial role in ligand binding in aqueous environments, we systematically varied the pH from 3 to 10 and used ^1^H NMR, ^31^P NMR, and DLS to assess the ligand binding and colloidal stability (Figure 6). From NMR, we extract the bound ligand fraction for the phosphonic acid ligands via peak deconvolution of the ^31^P resonances and for nitrodopamine-mPEG via peak deconvolution of the aromatic ^1^H resonances (Tables S3–S5). From DLS measurements, we obtained the *Z*-average value and the zeta-potential. The *Z*-average value is a single value describing the average particle size and is most sensitive to agglomeration. The zeta-potential indicates the degree of electrostatic repulsion between the nanocrystals (zeta-potential values above +25 mV or below −25 mV indicate stable suspensions). From Figure 6, we clearly observe that the bound ligand fraction decreases with increasing pH for both PA-PEG and PA-hex-PEG. Taking the p*K*_a_ values of ethyl phosphonic acid (p*K*_a__1_ = 2.43 ; p*K*_a__2_ = 8.05) as a reference, it is striking that the bound ligand fraction decreases most steeply around p*K*_a__2_. We infer that the second deprotonation of the phosphonic acid causes ligand–ligand repulsion in the ligand shell and increases the solubility of the ligand in water. Both effects lead to a reduced bound ligand fraction. Not surprisingly, the loss of ligands has a detrimental effect on the colloidal stability, and the *Z*-average increases significantly for pH > p*K*_a2_. Overall, PA-hex-PEG performs slightly better than PA-PEG, but the difference is small. Most likely, the double anionic phosphonate compensates for the short hydrophobic segment.

One might expect complete loss of colloidal stability upon ligand desorption. However, no visual turbidity was observed in the samples, and the *Z*-average remains below 100 nm. Note that the zeta-potential drops below −25 mV at pH >8. While steric stabilization is lost with progressing ligand desorption, electrostatic stabilization takes over, preventing the NCs from fully destabilizing. The negative charge could originate from residual-bound, double deprotonated phosphonates, or more likely from hydroxide adsorption on the nanocrystal surface. Indeed, in water, there are multiple adsorption–desorption equilibria present simultaneously.
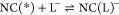
2
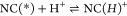
3
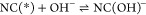
4In addition to these adsorption–desorption equilibria, there are also the acid–base equilibria, resulting in a highly complex pH dependence of the system.

According to Figure 6, the phosphonic acids keep the nanocrystals stable between pH 3 and pH 8 in a *static* system. However, in a dynamic biological environment (e.g., a blood vessel), many competing ligands are present and desorbed ligands are quickly removed. The equilibrium will adjust and thus ligands will continuously desorb from the surface, eventually causing loss of colloidal stability. Despite an acceptable *Z*-average value at physiological pH, this dynamic behavior could explain why single phosphonate ligands are not necessarily successful at fully preventing aggregation in physiological media.^62−66^

Interestingly, nitrodopamine-mPEG has almost the complete opposite behavior, unable to provide stable colloidal dispersions under acidic conditions with a very sharp transition around pH 5. This is evidenced by the steep increase in the *Z*-average (Figure 6). Between pH 5 and pH 10, all nitrodopamine-mPEG ligands remain bound, and only at pH 11, there is a slight decrease in the bound fraction from 100 to 97% (full range ^1^H NMR spectra in Figure S16). This translates to an excellent colloidal stability in the pH range of 5–11. We conclude that for aqueous applications at physiological pH, nitrodopamine-mPEG outperforms both PA-PEG and PA-hex-PEG. For aqueous applications at acidic pH, on the other hand, the phosphonic acid ligands are better suited. To test if there would also be a temperature dependence on the binding dynamics of the functionalized NC systems, we performed variable-temperature NMR measurements in D_2_O (pH 7.4) at 25, 37, and 60 °C (Figure S17). ^1^H NMR spectra showed no change in ligand adsorption/desorption equilibria for both phosphonic acids and nitrodopamine-mPEG, leading us to tentatively conclude that the results from the pH titrations would also translate to physiological temperature.

To illustrate the complementary behavior of phosphonic acids and catechols, we performed a competitive exchange reaction on purified nitrodopamine-mPEG NCs in D_2_O. One equivalent of PA-PEG was added, compared to the original amount added to functionalize the NCs with nitrodopamine-mPEG, and NMR measurements were performed at different pH values (Figure S18). Figure S18 clearly shows that, at acidic pH values, a partial exchange with PA-PEG occurs, evidenced by the appearance of sharp nitrodopamine-mPEG signals in the aromatic region and methylene triplet around 3 ppm. The nitrodopamine-mPEG NC suspension, which normally would fully destabilize below pH 5, remained colloidally stable at pH 2.22 due to the now mixed catechol–phosphonate ligand shell. The exchange equilibrium mostly shifts back toward nitrodopamine-mPEG as the pH moves toward neutral and basic values.

### Stability in Phosphate-Buffered Saline

**Figure 7:**
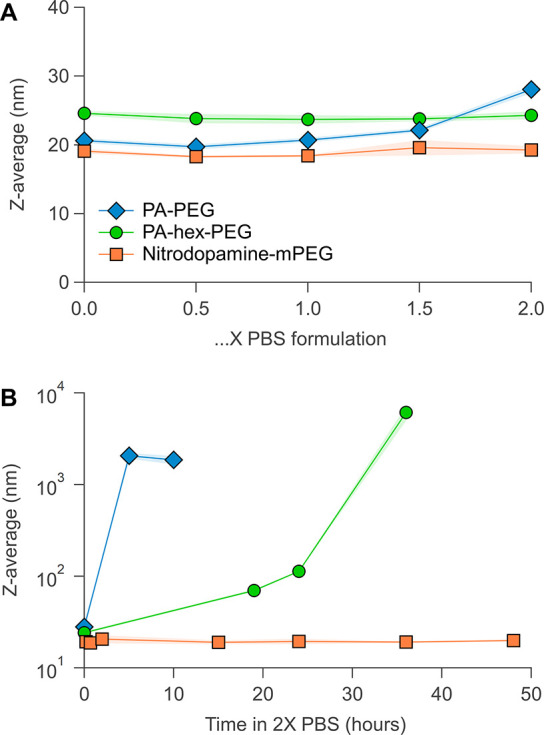
Stability of functionalized NCs in PBS at pH 7.4 and 25 °C. (A) Colloidal stability of functionalized nanocrystals measured using DLS *Z*-average values at different PBS concentrations. (B) Stability of functionalized NCs in 2× PBS over time.

Finally, we assessed the stability of NCs functionalized with PA-PEG, PA-hex-PEG, and nitrodopamine-mPEG in PBS. Stability in PBS is an important prerequisite for biomedical applications, as many in vivo experiments inject the desired drug or contrast agent in saline solution or in PBS. A standard 1× PBS buffer contains 137 mmol L^–1^ NaCl, 2.7 mmol L^–1^ KCl, 10 mmol L^–1^ Na_2_HPO_4_, and 1.8 mmol L^–1^ KH_2_PO_4_. When the concentrations are half or double, these buffers are referred to as 0.5× PBS and 2× PBS, respectively. It is clear that PBS contains relatively high salt concentrations and phosphate ions (which will compete for the surface) and thus provides a real test for the stability of our functionalized nanocrystals. First, we varied the PBS concentration and immediately measured the *Z*-average via DLS (Figure 7A). Up to 1.5× PBS, all ligands keep the nanocrystals colloidally stable. As expected, PA-PEG is the weakest ligand and cannot prevent the start of nanocrystal aggregation in 2× PBS solutions. Second, the stability of all functionalized NCs was monitored over time in 2× PBS (Figure 7B). Clearly, PA-PEG-functionalized nanocrystals agglomerate quickly and visually precipitate after several hours. The colloidal stability of PA-hex-PEG-functionalized nanocrystals steadily decreases over 24 h, before completely agglomerating. Only nitrodopamine-mPEG-functionalized nanocrystals remain perfectly stable. There is no sign of aggregation over the course of 48 h, and the suspension also remains visually clear for at least 1 month.

## Discussion

The above results make clear that surface chemistry becomes much more complex when moving from nonpolar to polar (e.g., aqueous) solvents. In nonpolar solvents, charged ligands or nanocrystals are thermodynamically unstable, leading to a limited set of binding motifs and clear ligand exchange rules.^25^ For example, autodesorption of oleate (deprotonated oleic acid) does not occur in toluene. For the binding motif of PbS(PbX_2_), removal of the entire Lewis acid PbX_2_ has been observed in chloroform or coordinating solvents like THF.^67^ Likewise, for HfO_2_(H,OOCR), desorption of oleic acid is possible by recombination of the carboxylate and proton.^22^ However, these restrictions disappear in polar solvents where charges are stabilized, and the proton and carboxylate have independent adsorption/desorption equilibria; see also eqs 2–4.

**Figure 8:**
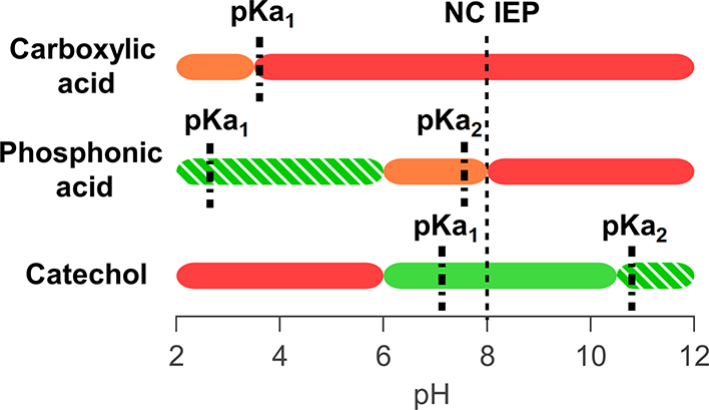
Colloidal stability map for metal oxide nanocrystals functionalized with carboxylic acids, phosphonic acids, and catechols. In this concrete example, we used HfO_2_ as the NC model system with its respective isoelectric point, MEEAA as the carboxylic acid, PA-PEG as the phosphonic acid, and nitrodopamine-mPEG as the catechol. The map colors should be interpreted as follows: green, excellent colloidal stability; striped green, high fraction of bound ligands but not perfect, use in a static system; orange, low bound ligand fraction, use in a static system without competition; red, nanocrystal aggregation/precipitation, do not use.

From the above data, we constructed a colloidal stability map, indicating which ligands provide colloidal stability in specific pH ranges (see Figure 8). It is interesting to correlate this stability map to the p*K*_a_ values of the ligands, which we experimentally determined via acid–base titrations (Figure S19). The p*K*_a_ values of PA-PEG are p*K*_a1_ = 2.65 ± 0.01 and p*K*_a2_ = 7.56 ± 0.01, and those of PA-hex-PEG are p*K*_a1_ = 2.99 ± 0.02 and p*K*_a2_ = 8.33 ± 0.01. At pH 3 (where the particles are stable), we calculate using eq 5 that approximately 70% of PA-PEG is monodeprotonated. One can assume that the phosphonate will bind in this form to the nanocrystal surface.
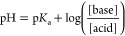
5For pH >8.5 (above p*K*_a2_), the bound ligand fraction decreases quickly and the particles start to aggregate (Figure 6). The double deprotonation of the ligand has two consequences; (1) the ligand becomes more soluble in water, and (2) electrostatic ligand–ligand repulsion appears, instead of the stabilizing hydrogen bonding. Both effects promote ligand desorption. In addition, the isoelectric point of HfO_2_ occurs at pH 8,^68^ rendering the surface negatively charged at pH >8, thus further decreasing the binding affinity of the negatively charged phosphonate ligand. Esarey et al. observed a similar phenomenon when studying the binding constant of a phosphonate-anchored rubidium complex on anatase TiO_2_ surfaces under varying pH conditions.^69^

**Figure 9:**
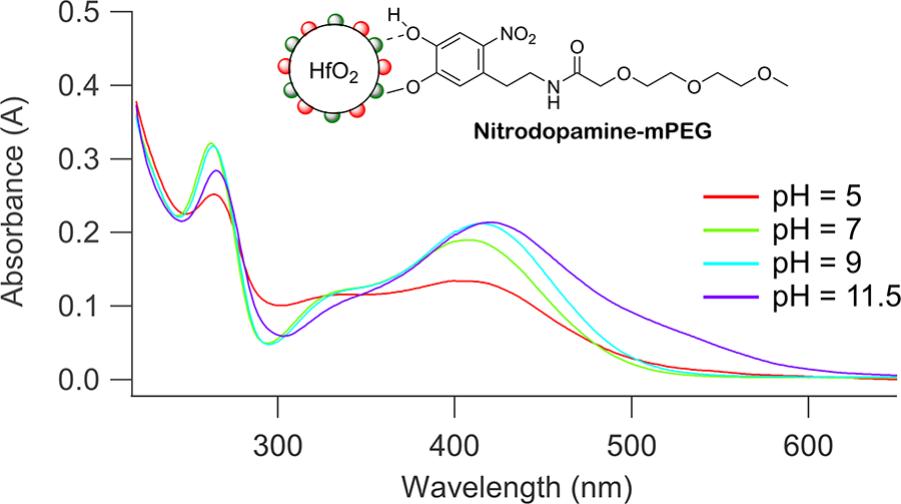
UV–vis spectra of purified nitrodopamine-mPEG-functionalized NCs at different pH values in H_2_O.

A similar reasoning applies to the case of nitrodopamine-mPEG (p*K*_a__1_ = 7.13 ± 0.01; p*K*_a__2_ = 10.8 ± 0.01). At pH = 5 (where the particles are unstable), we calculate that approximately 99% of the ligand is fully protonated and can thus only interact with the surface via weak hydrogen bonds.^70^ With increasing pH, a progressively higher fraction of nitrodopamine-mPEG is monodeprotonated, and is able to coordinate to the surface metal sites. It also provides an additional hydrogen bond to further stabilize the bound state. This is confirmed by comparing the UV–vis spectra of nitrodopamine-mPEG bound to HfO_2_ NCs with the reference spectra of the free ligand (see Figure 9 and Figure S20). For 5 < pH < 11, we find the monodeprotonated species. At pH >11, we see again the appearance of free ligands in NMR (Figure S16) and the double deprotonated species in UV–vis (Figure S20). For the reasons mentioned above, the double deprotonated species has a low binding affinity for the surface. In addition, the catechol becomes sensitive to oxidation at high pH. Finally, carboxylic acids can only be monodeprotonated and we observe that NCs functionalized with MEEAA (p*K*_a_ = 3.61 ± 0.03) gradually lose colloidal stability above pH 3.5 before fully precipitating above pH 6 (Figures S21 and S22). We hypothesize that they are less stable than phosphonates and catechols due to the lack of ligand–ligand hydrogen bonding.

The stability experiments in buffer solution point to an additional variable: competitive ligands. In phosphate-buffered saline, a high concentration of phosphate is present, which competes for the surface but does not provide steric stabilization. Since the phosphonic acids (PA-PEG and PA-hex-PEG) desorb at pH 7.4 (25% desorbed; see Figure 6), they are in equilibrium and can thus be slowly displaced over time. The nitrodopamine-mPEG is tightly bound (0% desorbed) and is not being displaced by phosphonic acid, judging from our competition experiments (Figure S18), hence the high stability of the particles capped with nitrodopamine-mPEG.

The above discussion shows that, in aqueous media, surface chemistry is a complex interplay between multiple factors. There is the pH-dependent charge of the surface, the pH-dependent deprotonation of the ligand, and competition by phosphate in buffer solution. Slight modifications to the ligand structure can alter the solubility of the ligands and thus the binding affinity. The binding group remains, however, the most important descriptor for the stability map, a conclusion supported by the relatively small differences between PA-PEG and PA-hex-PEG in their pH-dependent desorption from the surface. The colloidal stability map can thus be generalized to other carboxylate, phosphonate, and catecholate ligands. The p*K*_a_ values of the ligands used here are quite close to reported p*K*_a_ values for acetic acid (p*K*_a_ = 4.75), ethylphosphonic acid (p*K*_a__1_ = 2.43; p*K*_a__2_ = 8.05), and nitrodopamine (p*K*_a__1_ = 6.6; p*K*_a__2_ = 11).^71^ For optimal accuracy, it is recommended to insert the actual p*K*_a_ values for the ligands of interest, as the stable pH ranges will shift together with the p*K*_a_(s) of the ligand. For other oxide nanocrystals (e.g., TiO_2_ or Fe_2_O_3_), the corresponding isoelectric point needs to be used. Having thus the p*K*_a_(s) of the ligands available and the isoelectric point of the nanocrystals, one can now use the colloidal stability map (Figure 8) to start predicting which ligands will provide colloidal stability at a particular pH. A word of caution is warranted. Small structural variations in the ligand will have little influence on the predictions of the stability map, but significant structural variations, such as a high steric bulk, could have adverse effects on the binding affinity.

## Conclusion

We have mapped out the binding affinity of the three most important ligands for metal oxide nanocrystal surface functionalization. By performing an in-depth surface chemistry and stability study, we found that ligand dynamics become much more complex when moving from nonpolar to polar solvents. In water, the pH influences the ligand solubility through deprotonation of the binding group. Carboxylic acids are weakly binding anchors in both nonpolar and polar environments and can be easily displaced by competitive ligands. Phosphonic acids are strongly bound ligands in MeOH but desorb in aqueous media, the extent of which is dependent on the pH. They are mostly suited to stabilize nanocrystals in acidic environments. Finally, nitrocatechol derivatives provide a tightly bound ligand shell and excellent stability at physiological and basic pH. They are superior at stabilizing metal oxide nanocrystals in phosphate-buffered saline solutions but cannot be used in acidic environments. We have summarized our results in a convenient colloidal stability map that can be generalized to other oxides and ligands, provided the isoelectric points and p*K*_a_ values are available. This tool will allow researchers to rationally choose nanocrystal functionalization strategies for their desired applications in polar or aqueous environments.

## Experimental Methods

### Materials

(6-{2-[2-(2-Hydroxyethoxy)ethoxy]ethoxy}hexyl)phosphonic acid and (2-(2-(2-hydroxyethoxy)ethoxy)ethyl)phosphonic acid were purchased from SiKÉMIA. *N*-Hydroxysuccinimide (≥98%) and dopamine hydrochloride (≥99%) were purchased from Acros Organics. 2-[2-(2-Methoxyethoxy)ethoxy]acetic acid (>95.0%) was purchased from TCI Chemicals. Hafnium(IV) *tert*-butoxide (99.99%), *N*,*N*′-dicyclohexylcarbodiimide (99%), 4-(dimethylamino)pyridine (≥99%), 4-methylmorpholine (99%), sodium nitrite (≥99.0%), and solvents used for synthesis were purchased from Sigma-Aldrich. All purchased chemicals were used without further purification. All deuterated solvents were purchased from Sigma-Aldrich or Eurisotop.

### Synthesis of MEEAA-NHS

This procedure was adapted from Meissler et al.:^56^ 4 mmol (0.7128 g) 2-[2-(2-methoxyethoxy)ethoxy]acetic acid and 4.2 mmol (0.4828 g) *N*-hydroxysuccinimide were dissolved in 8 mL of dry THF in a predried vial and cooled to 0 °C. Then 4.2 mmol (0.866 g) *N*,*N*′-dicyclohexylcarbodiimide was dissolved in a separate predried vial in 4 mL of THF and added dropwise to the first mixture by performing an air-free transfer. The mixture was stirred for 15 min at 0 °C, after which 0.2 mmol (0.024 g) catalytic 4-dimethylaminopyridine was added. The mixture was stirred overnight at room temperature, resulting in a white turbid mixture. The turbid solution was filtered over a por 4 fritted glass filter, and the white precipitate was washed once with 10 mL of THF to collect the remaining product. After solvent removal of the filtrate using a rotary evaporator, the viscous liquid was dissolved in a minimum amount of DCM and transferred to a extraction funnel via a 0.2 μm syringe filter, and the organic phase was then diluted to a volume of 12 mL using DCM. The organic phase was extracted four times with 12 mL of Milli-Q (MQ) water and two more times with 12 mL of brine. The organic phase was dried over MgSO_4_ and dried using a rotary evaporator. Product was collected as a colorless to light yellow viscous liquid with 83% yield. ^1^H NMR (500 MHz, CDCl_3_): δ 4.5 (s, 2H), 3.8–3.76 (m, 2H), 3.7–3.66 (m, 2H), 3.65–3.6 (m, 2H), 3.55–3.51 (m, 2H), 3.36 (s, 3H), 2.82 (s, 4H). ^13^C NMR (500 MHz, CDCl_3_): δ 168.73 (s), 166.02 (s), 77.24 (s), 71.91 (s), 71.37 (s), 70.607 (s), 70.602 (s), 66.55 (s), 59.06 (s), 25.58 (s). HRMS 275.26 calcd for [M], 292.9 [M + NH_4_]^+^ found.

### Synthesis of Nitrodopamine Hemisulfate

The procedure was adapted from Napolitano et al.:^55^ 8.753 mmol (1.66 g) dopamine hydrochloride and 35.219 mmol (2.43 g) NaNO_2_ were dissolved in 100 mL of MQ water and cooled in an ice bath. Then 8.33 mL of precooled 20% H_2_SO_4_ was added dropwise to the mixture under heavy stirring; during addition, the mixture turned turbid yellow with the formation of brown gases. The mixture was removed from the ice bath and was allowed to stir 12 h at room temperature. The resulting turbid yellow solution was cooled again in an ice bath, followed by collection of the solid via suction filtration using a por 4 fritted glass filter. Next, the solid was washed two times with 50 mL of ice-cold MQ water, one time with 50 mL of ice-cold absolute ethanol, and two times with 50 mL of ice-cold diethyl ether. The yellow powder was collected and dried overnight under vacuum, final yield was 50%. ^1^H NMR (500 MHz, DMSO-*d*_6_): δ 7.46 (s, 1H), 6.73 (s, 1H), 3.12–2.99 (m, 4H). ^13^C NMR (500 MHz, DMSO-*d*_6_): δ 156.49 (s), 145.24 (s), 136.31 (s), 127.32 (s), 111.14 (s), 39.15 (s), 31.56 (s). HRMS 296.25 calcd for [M], 197.00 [M – H_2_SO_4_ – H]^−^ found.

### Synthesis of Nitrodopamine-mPEG

The procedure was inspired by Amstad et al.:^54^ 1.784 mmol (491 mg) MEEAA-NHS and 2.854 mmol (846 mg) nitrodopamine hemisulfate were dissolved in 25 mL of dry DMF in a predried flask, resulting in a dark orange solution. The flask was sealed, flushed with argon, and cooled in an ice bath. Then 785 μL of *N*-methylmorpholine was added dropwise to the mixture using air-free technique; after approximately 15 min of stirring, the solution became turbid. The mixture was allowed to stir 48 h at room temperature, followed by evaporation overnight under vacuum at 40 °C to remove DMF, yielding a dark brown liquid. Next, 40 mL of 1 M HCl was added to the crude and extracted three times with 40 mL of CHCl_3_; a dark brown solid is formed during the process at the liquid interface, and care was taken to not allow this to enter the organic phase. The organic phase was extracted with 50 mL of brine, dried with Na_2_SO_4_, and evaporated using rotary evaporation. The resulting solid was purified with preparative HPLC using a gradient from solvent A (MQ water containing 0.1% TFA) to solvent B (ACN containing 0.1% TFA), and after being freeze-dried, the final product was isolated as a fluffy white-yellow solid with 75% yield. ^1^H NMR (500 MHz, MeOD): δ 7.54 (s, 1H), 6.7 (s, 1H), 3.93 (s, 2H), 3.62 (s, 4H), 3.61–3.58 (m, 2H), 3.57–3.5 (m, 4H), 3.35 (s, 3H), 3.07 (t, 2H, *J* = 6.68 Hz). ^13^C NMR (500 MHz, MeOD): δ 173.05 (s), 152.4 (s), 145.53 (s), 141.06 (s), 129.39 (s), 119.61 (s), 113.56 (s), 72.97 (s), 72.07 (s), 71.4 (s), 71.39 (s), 71.24 (s), 59.21 (s), 40.47 (s), 34.14 (s). HRMS 358.35 calcd for [M], 357.35 [M – H]^−^ found.

### Synthesis of Hafnium Oxide Nanocrystals

The NCs were synthesized from hafnium(IV) *tert*-butoxide (5.47 mmol, 2.58 g, 2.21 mL) and anhydrous benzyl alcohol (45.6 mL) according to Lauria et al.^48^ After the synthesis, the nanocrystals were collected by adding diethyl ether (19 mL) to the reaction mixture and centrifugation (5000 rcf, 5 min) in plastic centrifuge tubes. The sediment was washed three times with diethyl ether (19 mL). For functionalization with 2-(2-(2-methoxyethoxy)ethoxy)acetic acid, the sediment was first dispersed in 19 mL of toluene, resulting in a milky white turbid liquid. Then 381 μL of 2-(2-(2-methoxyethoxy)ethoxy)acetic acid (0.328 g; 1.82 mmol) was added followed by 30 min of sonication, resulting in a transparent suspension with a few insolubles present. The insolubles were removed by centrifugation (5000 rcf, 5 min), and the clear top layer was transferred to new plastic centrifuge tubes. NCs were precipitated by addition of 1:2 volume of hexane (mixture of isomers); after centrifugation (5000 rcf, 5 min), the organic top phase was removed, and the NCs were resuspended in toluene. This purification step was repeated three more times before final resuspension in toluene, and the final NC yield was 70%. The purified NC suspension in toluene remains stable for at least 1 year. The dispersion in toluene can be dried and dispersed in ethanol. From ethanol, the dispersion can be dried again and resuspended in MeOH, and from MeOH, the dispersion can be dried again and resuspended in H_2_O.

## Supporting Information Available

Additional experimental methods, NMR spectra of titrations and synthesized compounds, PDF analysis, peak deconvolutions, UV–vis spectra of nitrodopamine-mPEG, DLS data of carboxylate-capped nanocrystals; acid-base titrations for all ligands (PDF)
