# Supplementary material for: Mapping out the Aqueous Surface Chemistry of Metal Oxide Nanocrystals: Carboxylate, Phosphonate, and Catecholate Ligands
Source: JACS Au. 2022 Mar 4;2(3):711–22. doi: 10.1021/jacsau.1c00565 (PMC8969999; doi:10.1021/jacsau.1c00565)
Supplement: Supplementary file 1 — au1c00565_si_001.pdf [file au1c00565_si_001.pdf]

Supporting information

for

Mapping out the Aqueous Surface Chemistry of Metal Oxide Nanocrystals: Carboxylate, Phosphonate, and Catecholate Ligands

Loren Deblock;[1,2] Eline Goossens,[1] Rohan Pokratath,[2] Klaartje De Buysser,[1] Jonathan De Roo[2]*

[1] Department of Chemistry, Ghent University, 9000 Ghent, Belgium

[2] Department of Chemistry, University of Basel, 4058 Basel, Switzerland

Corresponding author: Jonathan De Roo, Jonathan.deroo@unibas.ch

Contents

1. Experimental details 2
2. Supporting figures 8
3. Spectra of synthesized compounds 23
4. ^{31}P peak deconvolution - D_2O titration 29
5. ^{31}P and ^{1}H peak deconvolution - pH titration 31

1. Experimental details

TEM analysis. Transmission electron microscopy (TEM) images (of a drop-cast suspension on a grid) were taken on a JEOL JEM-2200FS TEM with Cs corrector and a JEOL JEM-1010 operating at 100 kV.

Dynamic light scattering analysis. Dynamic light scattering (DLS) and Zeta potential measurements were conducted on a Malvern Zetasizer Ultra Dynamic Light Scattering system in backscattering mode (173°). DLS and Zeta potential measurements were performed respectively in glass cuvettes and disposable folded capillary cells. All measurements were performed in triplicate at 25 °C after equilibrating inside the system for 240 seconds, sample concentration was tuned to achieve system attenuator values between 9-10. DLS data processing was performed using the Malvern "ZS Explorer" software using the "general purpose" analysis model, Zeta potential data processing was performed in the same software using the "monomodal" analysis model.

UV-Vis analysis. UV-Vis spectra were recorded on a PerkinElmer Lambda 365.

X-ray total scattering and pair distribution function analysis. The Pair Distribution Function (PDF) measurement was conducted at beamline P21.1 at DESY in Hamburg, Germany in rapid acquisition mode, using a Varex 2D detector (2880 × 2880 pixels and 150 × 150 µm pixel size) with a sample to detector distance of 800 mm. The incident wavelength of the X-rays was $\lambda = 0.122$ Å. Calibration of the experimental setup was performed using a nickel standard. Raw 2D data were corrected for geometrical effects and polarization, then azimuthally integrated to produce 1D scattering intensities versus the magnitude of the momentum transfer Q (where $Q = 4\pi \sin \theta/\lambda$ for elastic scattering) using pyFAI and xpdtools.[1, 2] xPDFsuite with PDFgetX3 was used to perform the background subtraction, further corrections, and normalization to obtain the reduced total scattering structure function $F(Q)$ and Fourier transformation to obtain the PDF, $G(r)$.[3, 4] Modeling was carried out using Diffpy-CMI.[5]

Variable temperature NMR measurements. Variable temperature ^{1}H NMR measurements were recorded on a Bruker Avance III NMR spectrometer operating at 600.13 MHz proton frequency, the instrument was equipped with an indirect 5-mm BBI probe. The probe is provided with self-shielded z-gradients. For experiments performed below 318 K the temperature was calibrated using a methanol standard showing accuracy within +/- 0.2 K. For variable temperature NMR measurements above 318 K a glycerol standard was used for calibration.

NMR measurements on PA-PEG functionalized NCs. Nuclear magnetic resonance (NMR) measurements for nanocrystal (NC) functionalizations with (2-(2-(2-hydroxyethoxy)ethoxy)ethyl)phosphonic acid (PA-PEG) were recorded on a Bruker Avance III NMR spectrometer (titration with PA-PEG and transfer to water) operating at 600.13 MHz proton frequency. The instrument was equipped with a direct observe 5-mm BBFO smart probe (for ^{31}P NMR) or with an indirect 5-mm BBI probe. Both probes are provided with self-shielded z-gradients. The experiments were performed at 298 K and the temperature was calibrated using a methanol standard showing accuracy within +/- 0.2 K.

All other ^{1}H NMR measurements for functionalization with PA-PEG were recorded at a temperature of 298 K on a Bruker Avance III HD NMR spectrometer operating at 600.13 MHz proton frequency, the instrument was equipped with a cryogenic QCI-F probe. All other ^{31}P NMR measurements for functionalization with PA-PEG were recorded at a temperature of 298 K on a Bruker Avance Neo spectrometer operating at 500.13 MHz proton frequency, the instrument was equipped with a BBFO probehead. Probes for both spectrometers are provided with self-shielded z-gradients. The temperature was calibrated using a methanol standard showing accuracy within +/- 0.2 K.

NMR measurements on PA-hex-PEG and nitrodopamine-mPEG functionalized NCs. Nuclear magnetic resonance (NMR) measurements for nanocrystal (NC) functionalizations with (6-(2-(2-(2-hydroxyethoxy)ethoxy)ethoxy)hexyl)phosphonic acid (PA-hex-PEG) and N-(4,5-dihydroxy-2-nitrophenethyl)-2-(2-(2-methoxyethoxy)ethoxy)acetamide (nitrodopamine-mPEG) were recorded on a Bruker Avance III HD NMR spectrometer operating at 600.13 MHz proton frequency, the instrument was equipped with a cryogenic QCI-F probe. ^{31}P NMR measurements for functionalization with PA-hex-PEG were recorded at a temperature of 298 K on a Bruker Avance Neo spectrometer operating at 500.13 MHZ proton frequency, the instrument was equipped with a BBFO probehead. Probes for both spectrometers are provided with self-shielded z-gradients. The temperature was calibrated using a methanol standard showing accuracy within +/- 0.2 K.

NMR measurements on synthesized ligands. ^{1}H, ^{13}C{^{1}H}, ^{31}P{^{1}H} and 2D NMR measurements for synthesized ligands were recorded at a temperature of 298 K on a Bruker Avance Neo spectrometer operating at 500.13 MHz proton frequency, the instrument was equipped with a BBFO probehead. The probe is provided with self-shielded z-gradients. The temperature was calibrated using a methanol standard showing accuracy within +/- 0.2 K.

NMR experimental parameters. For quantitative 1D ^{1}H measurements, 64k data points were sampled with the spectral width set to 20 ppm and a relaxation delay of 30s. Concentrations were obtained using the digital ERETIC method.[6] DOSY measurements were performed with a double stimulated echo and bipolar gradient pulses (dstebpgp2s). The gradient strength was varied quadratically from 2-95% of the probe's maximum value in 8 steps if a diffusion filtered slice was required or 32 steps if the creation of a pseudo 2D spectrum was required. The gradient pulse duration and diffusion delay were optimized to ensure a final attenuation of the signal in the final increment of less than 10% relative to the first increment. The diffusion coefficients were obtained by fitting a modified Stejskal-Tanner equation to the signal intensity decay:

$$\mathrm{I} = \mathrm{I}_0 e^{-(\gamma\delta g)^2 \mathrm{D}(\Delta - 0.6\delta)}$$

I are the signal intensities, D are the linear diffusion coefficients, γ is the gyromagnetic ratio of the studied nucleus, g is the gradient strength, δ is the pulsed field gradient duration and Δ is the diffusion delay. A correction factor of 0.6 is applied for δ due to the smoothed squared pulse shape used for the gradient pulses. For 1D $^{31}P\{^{1}H\}$ measurements, in the zgpg30 pulse sequence 25000 data points were sampled with the spectral width set to 270.81 ppm and 4k scans, LB was set to 40 Hz during spectrum post-processing. For 1D $^{13}C\{^{1}H\}$ measurements, in the zgpg30 pulse sequence 120480 data points were sampled with the spectral width set to 239.49 ppm and 4k scans.

Spin filtration purification in MeOH and H_2O. An NC suspension containing maximum 50 mg material dissolved in 2 ml solvent is transferred to a pre-rinsed Sartorius Vivaspin (30000 MWCO) spin-filtration tube via a 0.2 µm syringe filter. The suspension is diluted to a volume of 20 ml with MeOH or Milli-Q water, the solution was then allowed to spin in a centrifuge for 30 mins at 2100 rcf. For phosphonic acid NC functionalizations 3 spin filtration cycles were performed per sample using MeOH, for nitrodopamine-mPEG NC functionalizations a minimum of 2 spin filtration cycles using Milli-Q water were performed until the filtrate was colorless. The concentrate was collected, evaporated and suspended in (deuterated) MeOH or (deuterated) H_2O, 30 minutes of sonication was performed to ensure that all agglomerates were resuspended and to minimize insolubles.

Titration with PA-PEG and PA-hex-PEG. A small amount of the purified toluene NC stock suspension was evaporated to yield approximately 45 mg functionalized material. The NCs were suspended in 0.5 ml absolute EtOH and sonicated for 30 mins, after which the solvent was evaporated again. The NCs

were then suspended in 0.5 ml MeOD and sonicated for 30 mins, a quantitative ^{1}H NMR measurement was performed using the digital ERETIC method to determine the MEEAA concentration. Care was taken to subtract the MeOH solvent peak, which partly overlaps with MEEAA signals, from the calculation to ensure accurate concentration determination. Next, a stock solution containing at least 3 equivalents of either PA-PEG or PA-hex-PEG was created in MeOD. The titration was performed by addition of PA-PEG or PA-hex-PEG in steps of 0.1 equivalents, at each addition step the NMR tube was flipped, then mixed using vortex rotation for 2 minutes followed by sonication for a few seconds.

NC functionalization with PA-PEG and PA-hex-PEG. In a typical functionalization the same method as during the titration with PA-PEG or PA-hex-PEG is used. Except here 1.5 equivalents of phosphonic acid are added all at once to the MEEAA functionalized NCs, stirred and sonicated 10 mins, followed by purification using spin filtration to yield pure PA-PEG or PA-hex-PEG functionalized NCs.

Titration with D_2O. NCs functionalized with PA-PEG or PA-hex-PEG were purified using spin filtration as described above, the concentrate was evaporated and resuspended in 500 µL MeOD. D_2O was added in a stepwise manner to achieve a final D_2O concentration of 25, 50, 75 and 100% respectively. When required, the suspension was evaporated between measurements to achieve the desired D_2O concentration without increasing sample volume above 0.8 ml.

Titration with nitrodopamine-mPEG. A small amount of the purified toluene NC stock suspension was evaporated to yield approximately 10 mg functionalized material. The NCs were suspended in 0.5 ml absolute EtOH and sonicated for 30 mins, after which the solvent was evaporated again. The NCs were then suspended in 0.5 ml MeOH and sonicated for 30 mins, after which the solvent was evaporated again. The NCs were suspended in 0.5 ml D_2O and a quantitative ^{1}H NMR measurement was performed using the digital ERETIC method to determine the MEEAA concentration. 1.5 equivalents of nitrodopamine-mPEG (compared to the amount of MEEAA on the NCs) was preactivated in D_2O by addition of 2 equivalents of NaOD (compared to the amount of nitrodopamine-mPEG required). Preactivated nitrodopamine-mPEG was added in steps of 0.5 equivalents while ensuring that pH remains above 5 during the addition, after each addition step the NMR tube was flipped, then mixed using vortex rotation for 2 minutes followed by sonication for a few seconds.

NC functionalization with nitrodopamine-mPEG. In a typical functionalization the same method as during the titration with nitrodopamine-mPEG is used. Except here 1.5 equivalents of preactivated

nitrodopamine-mPEG are added all at once while ensuring the pH remains above 5 during the entire addition. The mixture was stirred and sonicated 10 mins, followed by purification using spin filtration to yield pure nitrodopamine-mPEG functionalized NCs. The authors note that this method is scalable to higher amounts of NCs as long as the maximum loading allowed per spin filter is not exceeded.

Influence of pH on ligand binding. For all measurements 2 ml solvent was found to be the minimum required for the micro pH electrode to be able to measure pH values. A 5M NaCl stock solution was used to achieve a sample salt concentration of 0.01M, pH values were adjusted using 0.01M stock solutions of DCl and NaOD in D_2O.

Phosphonic acids: pure PA-PEG and PA-hex-PEG functionalized NCs in MeOH were created using above methods, the NC suspension was evaporated and resuspended in D_2O. ^{31}P NMR measurements were performed at several pH values. The amount of bound and unbound ligands in the ^{31}P spectra were quantified via a multi-peak fitting procedure (peak deconvolution).

Nitrodopamine-mPEG: pure nitrodopamine-mPEG functionalized NCs in Milli-Q water were created using above methods, the NC suspension was evaporated and resuspended in D_2O. Quantitative ^{1}H NMR measurements were performed at several pH values. The amount of bound and unbound ligands in the ^{1}H spectra were quantified via a multi-peak fitting procedure (peak deconvolution).

Dynamic light scattering stability evaluation. For all measurements pure functionalized NCs in MeOH (for phosphonic acids) or Milli-Q water (for nitrodopamine-mPEG) were created using above methods, the NC suspensions were evaporated and resuspended in Milli-Q water. For Z-average and zeta potential measurements NC concentration was tuned to achieve system attenuator values between 9-10. 2 ml solvent was found to be the minimum required for the micro pH electrode to be able to measure pH values and to perform Z-average and zeta potential measurements. All measurements were performed in triplicate at 25 °C after equilibrating inside the system for 240 seconds.

Influence of pH on Z-average values and zeta potential. A filtered 5M NaCl stock solution was used to achieve a sample salt concentration of 0.01M, the suspensions were sonicated for 15 minutes and filtered through a 0.2 µm Supor syringe filter to remove dust before starting the titration. pH values were adjusted using 0.01M filtered stock solutions of HCl and NaOH in Milli-Q water.

Stability in 2X PBS: The suspension was filtered through a 0.2 µm Supor syringe filter, followed by pH adjustment to 7.4 using 0.01M filtered stock solutions of HCl and NaOH in Milli-Q water. PBS

concentration was increased by stepwise addition of a filtered 10X PBS stock solution, pH was checked after each addition step and readjusted to 7.4 if required. Stability over time in 2X PBS measurements were performed in closed quartz cuvettes, which remained at room temperature and in closed conditions during the entire duration of the stability tests.

pKa determination for all ligands. pKa determination of all ligands was performed via acid-base titrations in water using a Hanna Instruments automatic potentiometric titrator (HI931-02). The instrument was first calibrated via a three-point calibration using buffers at pH 4.01, 7.01 and 10.01. All titrations were performed in triplicate using an aqueous 0.1 mol.L^{-1} NaOH solution as titrant. Titrations for MEEAA, PA-PEG and PA-hex-PEG were performed using 50 ml ligand solution per titration with ligand concentration at approximately 0.01 mol.L^{-1}. Titrations with nitrodopamine-mPEG were performed using 50 ml ligand solution per titration but at a lower concentration due to limitations in the amount of ligand that was available, concentrations here were approximately 0.003 mol.L^{-1}, NaOH addition volumes were minimized in this case to compensate for the lower ligand concentration. Equivalence points for each titration curve were determined by plotting the first derivative of the titration curve in function of the midpoint volume of NaOH added, the maxima of the first derivative curve indicate the equivalence points. Through knowledge of the equivalence points the pKa values can then also be extracted from the curve.

2. Supporting figures

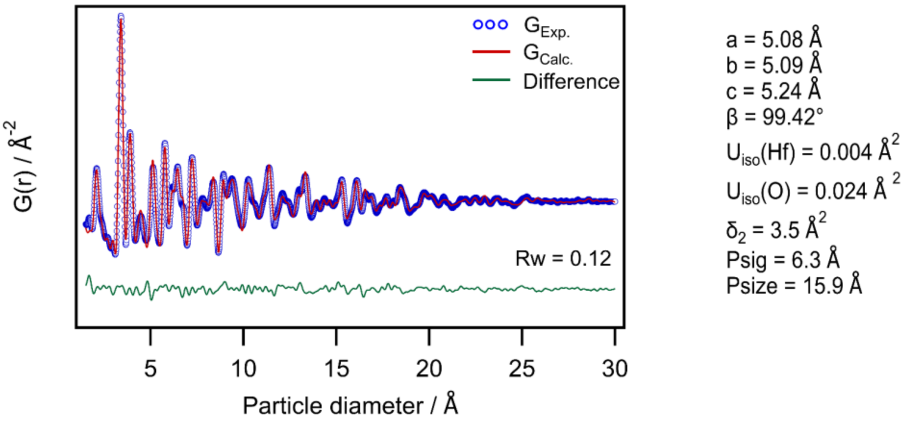

Figure S1: Pair Distribution Function (PDF) fit for the hafnium oxide nanocrystals with monoclinic ($P2_1/c$) phase. Refinement was carried out with lognormal particle size distribution. Refined parameters are indicated.

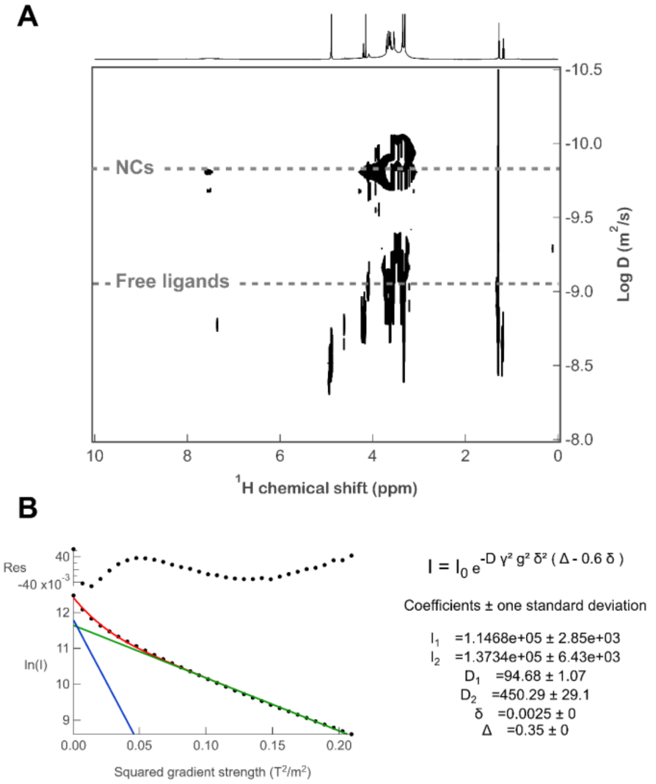

Figure S2: Diffusion Ordered Spectroscopy (DOSY) data from MEEAA functionalized HfO_2 nanocrystals in MeOD. **A**: 2D DOSY spectrum. A clear distinction can be seen between the slow moving signals of ligands bound to the nanocrystal surface and freely diffusing ligands. **B**: Intensity decay curve fitted using a modified Stejskal-Tanner equation and bi-exponential fit function. I are the signal intensities, D are the linear diffusion coefficients, γ is the gyromagnetic ratio of the studied nucleus, g is the gradient strength, δ is the pulsed field gradient duration and Δ is the diffusion delay. A correction factor of 0.6 is applied for δ due to the smoothed squared pulse shape used for the gradient pulses.

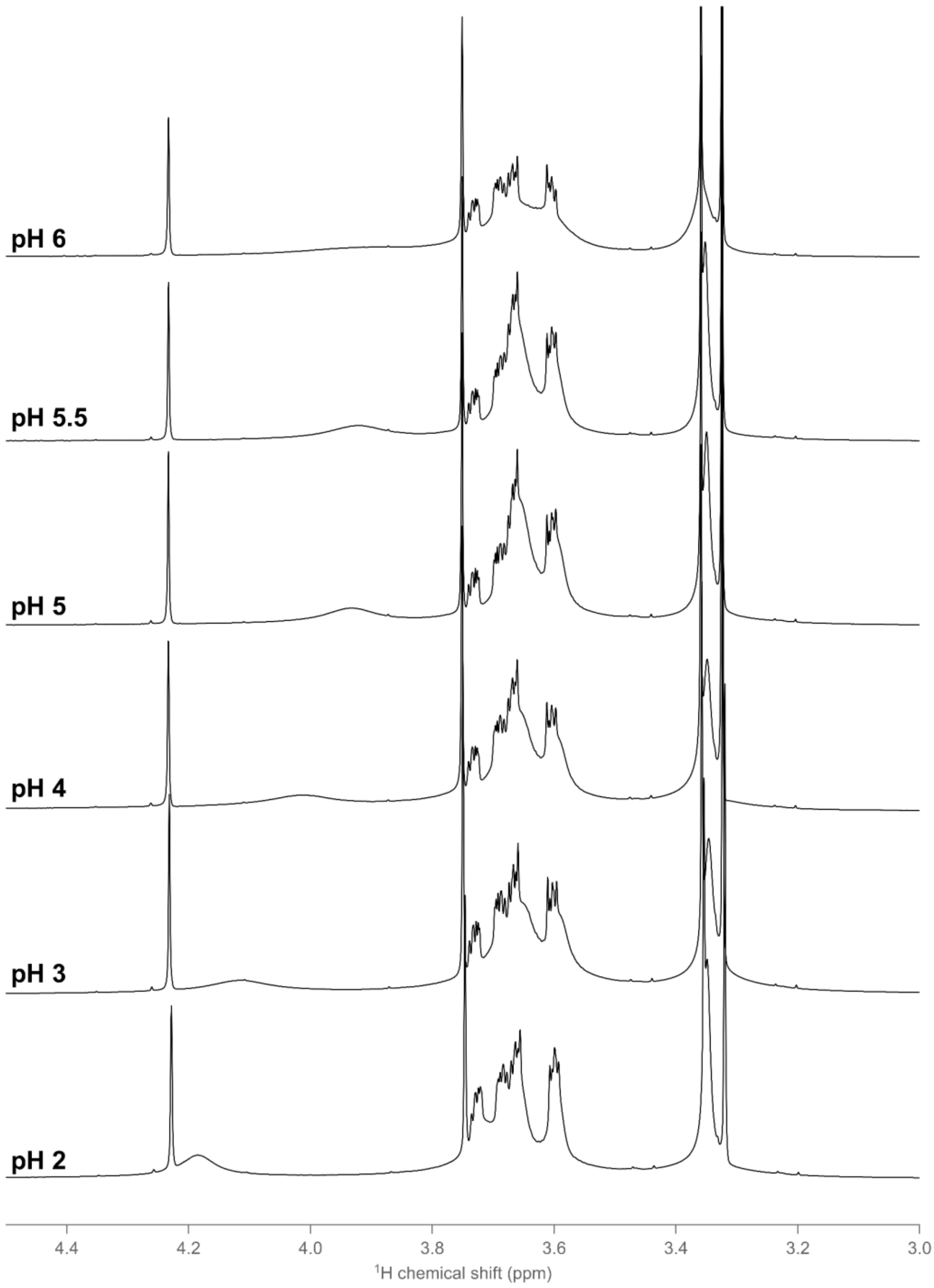

Figure S3: ^{1}H NMR spectra of the MEEAA functionalized NCs at different pH values in D_2O, the titration was stopped at pH 6 because the NCs sedimented out above this pH value. 4.4-3.8 ppm contains two signals both arising from the protons residing on the alfa carbon, the sharp signal that remains stationary originates from freely diffusing MEEAA, the broader peak that shifts as pH becomes more basic originates from MEEAA molecules that are in a dynamic equilibrium with the surface. 3.8 – 3.2 ppm contains the MEEAA ethoxy and terminal methyl proton signals. pH adjustments were made using DCl and NaOD solutions in D_2O.

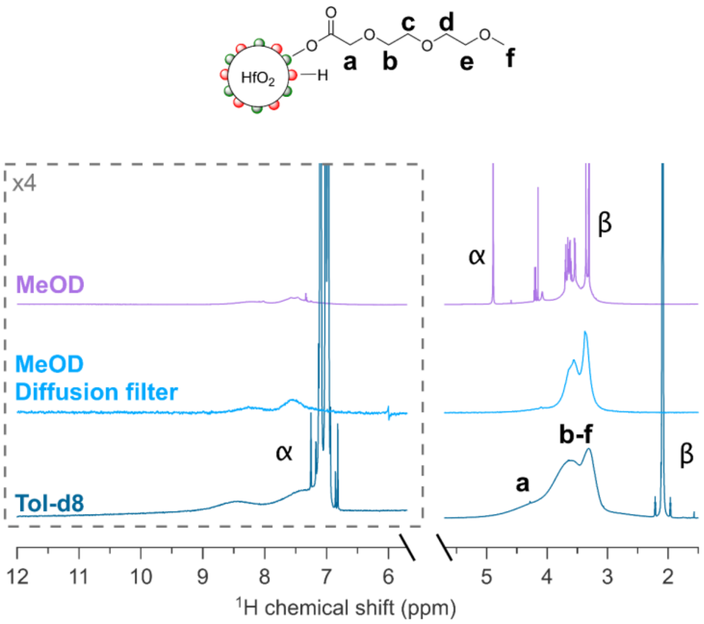

Figure S4: Full range (diffusion filtered) ^{1}H NMR spectra of MEEAA functionalized HfO_2 NCs in different solvents. A multiplication factor was applied in the 5.5-12 ppm region for easier data interpretation.

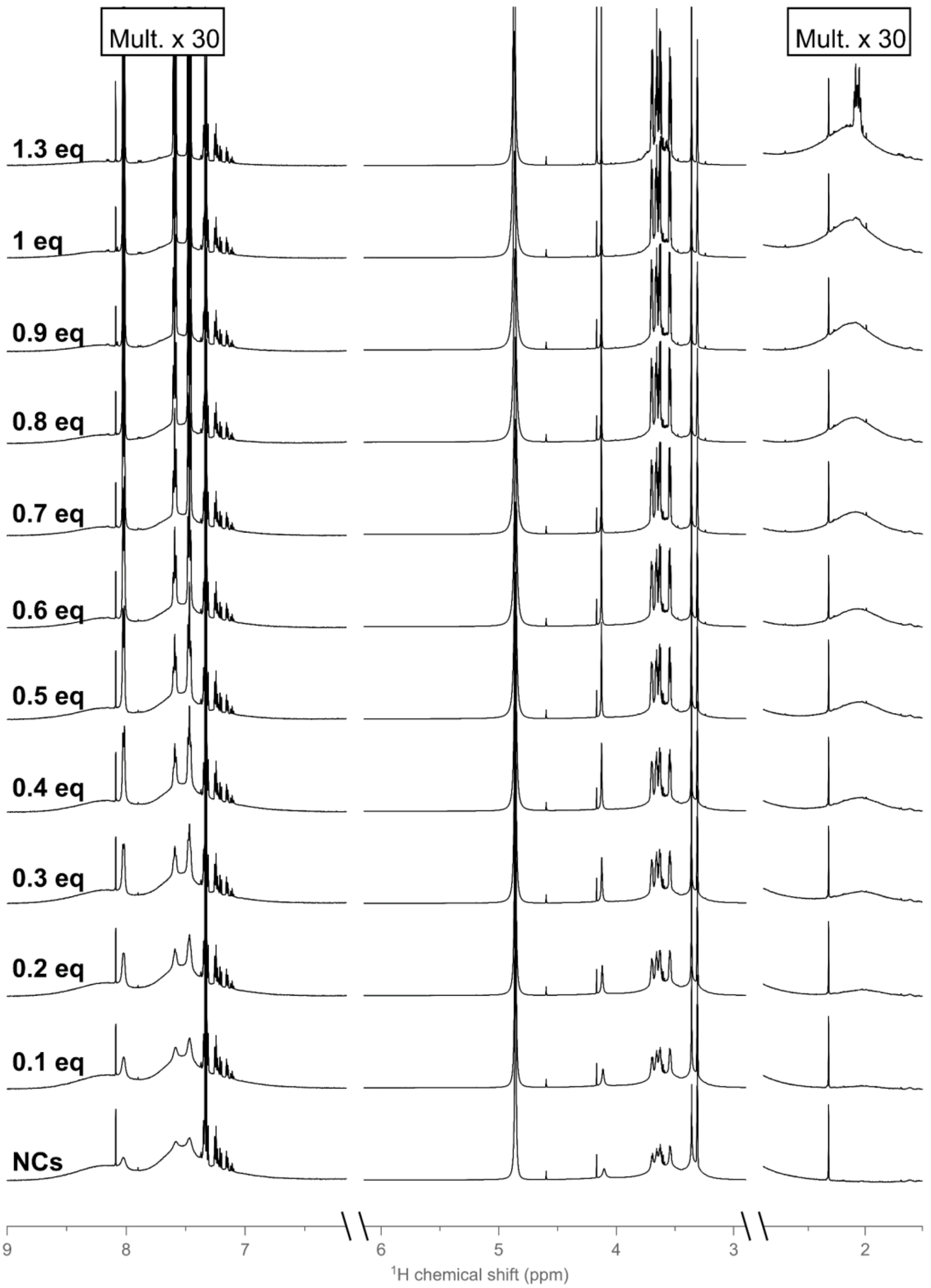

Figure S5: Full range ^{1}H NMR spectra of the MEEAA functionalized NC titration with the PA-PEG ligand in MeOD, the graph has been split in 3 parts each with their own multiplication factor for easier data interpretation. 3 distinct areas can be distinguished: 6.5-9 ppm contains aromatic signals arising from the formation of benzoic acid during NC synthesis using benzyl alcohol as solvent, 3-5 ppm contains signals arising from the PEG ethoxy moieties, finally 1-2.5 ppm contains the 'fingerprint' region of PA-PEG, an isolated signal arising from the protons on the ligand alfa carbon, the sharp signal at 2.4 ppm arose from a solvent impurity.

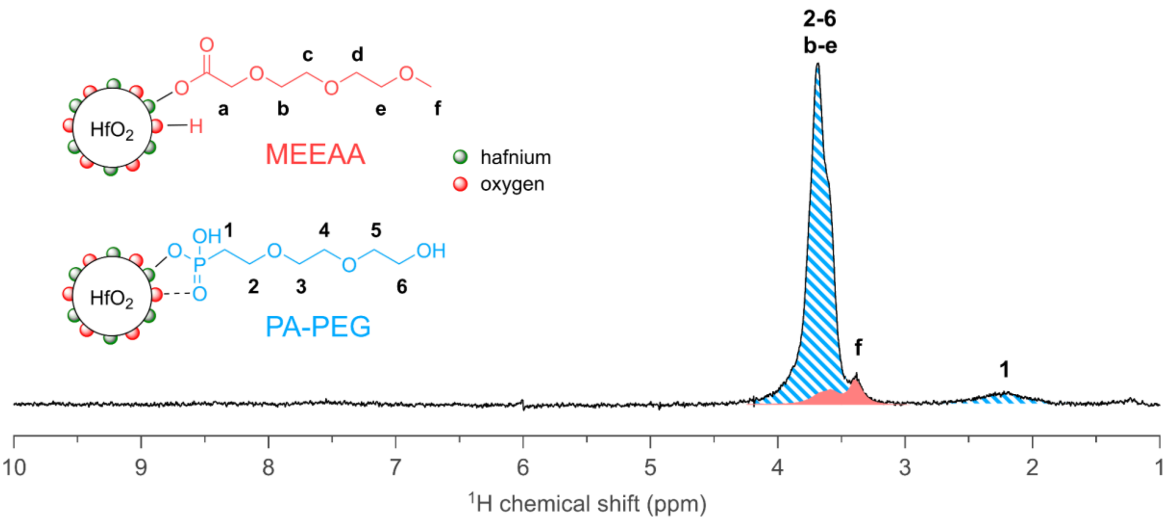

Figure S6: Full range diffusion filtered ^{1}H NMR spectra of MEEAA functionalized NCs in MeOD after addition of 1.3 equivalents of PA-PEG. Signals arising from bound MEEAA are denoted in red, signals arising from PA-PEG are denoted in striped blue.

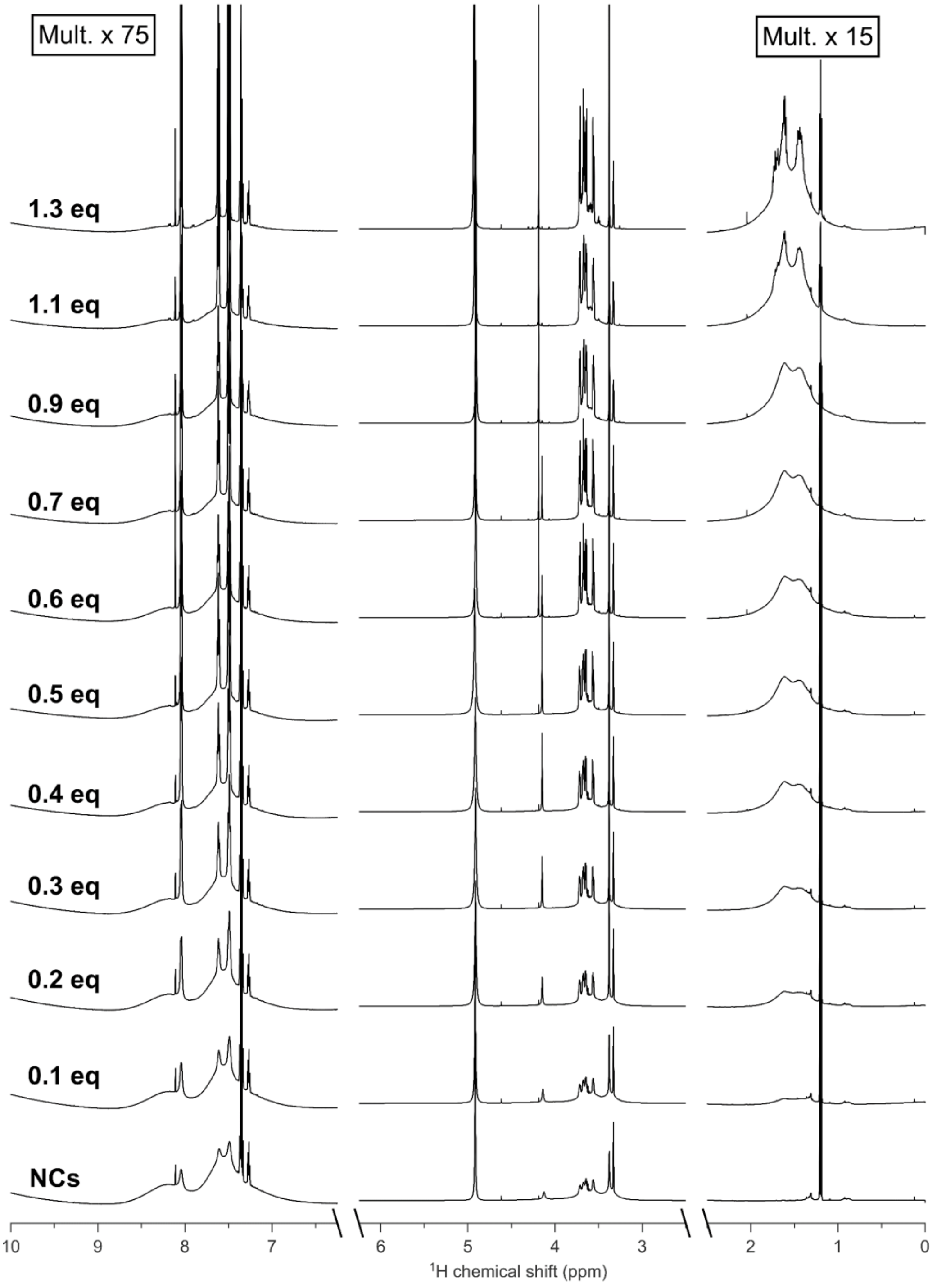

Figure S7: Full range ^{1}H NMR spectra of the MEEAA functionalized NC titration with the PA-hex-PEG ligand in MeOD, the graph has been split in 3 parts each with their own multiplication factor for easier data interpretation. 3 distinct areas can be distinguished: 6.5-9 ppm contains aromatic signals arising from the formation of benzoic acid during NC synthesis using benzyl alcohol as solvent, 3-5 ppm contains signals arising from the PEG ethoxy moieties and one aliphatic CH_2, finally 1-2.5 ppm contains the 'fingerprint' region of PA-hex-PEG, arising from the protons on carbon 1-5 in the ligands aliphatic hexyl chain. The sharp signal around 1.2 ppm arose from a solvent impurity.

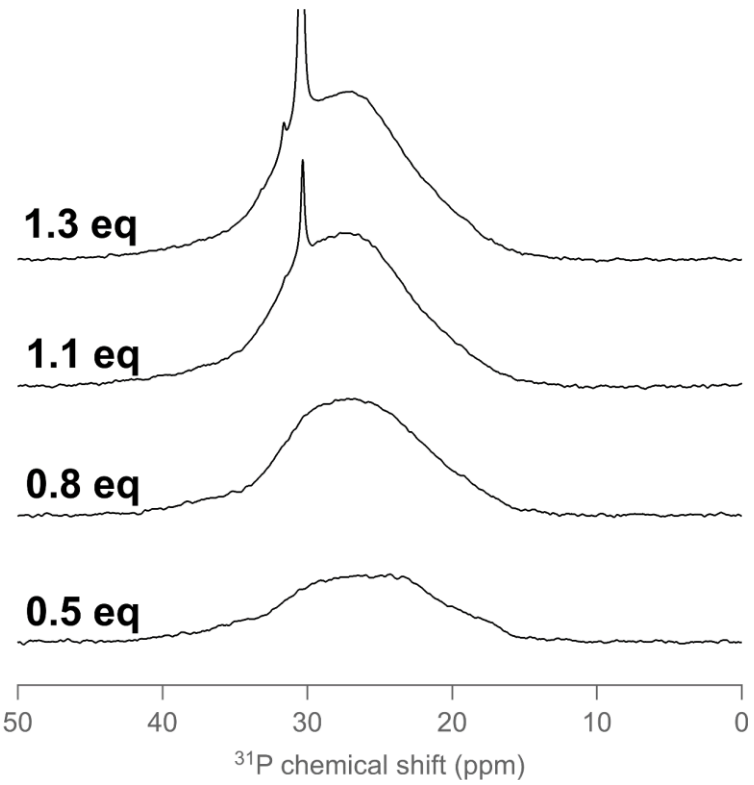

Figure S8: ^{31}P NMR spectra of the MEEAA functionalized NC titration with the PA-hex-PEG ligand in MeOD, at 1.1 equivalents added a sharp unbound ligand peak is observed. As before with PA-PEG the bound ligand peak slightly increases in intensity between 1.1 and 1.3 equivalents despite there being unbound ligands present in solution.

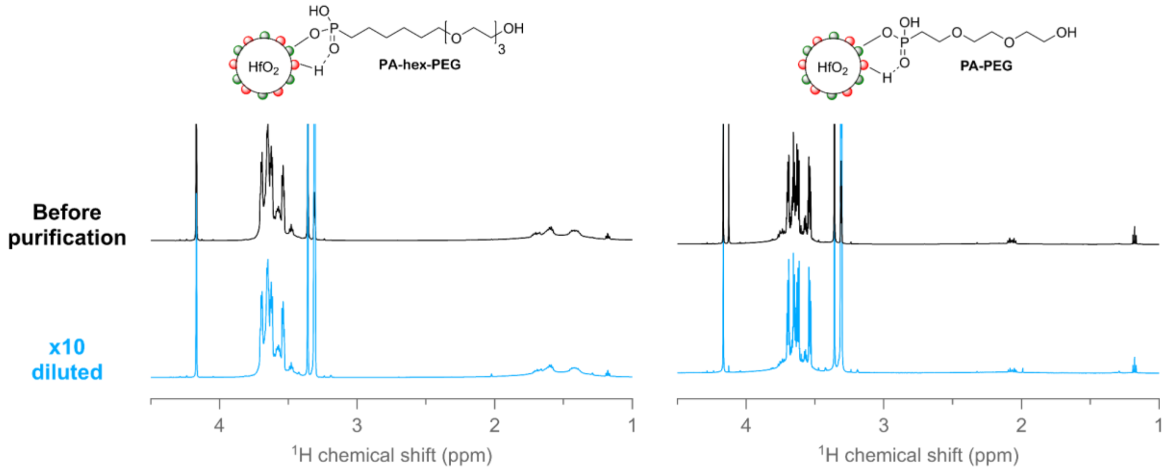

Figure S9: ^{1}H NMR spectra of PA-PEG and PA-hex-PEG functionalized NCs before purification and after dilution with MeOH. No shift in ligand the adsorption/desorption equilibria can be observed from dilution of the NC suspensions.

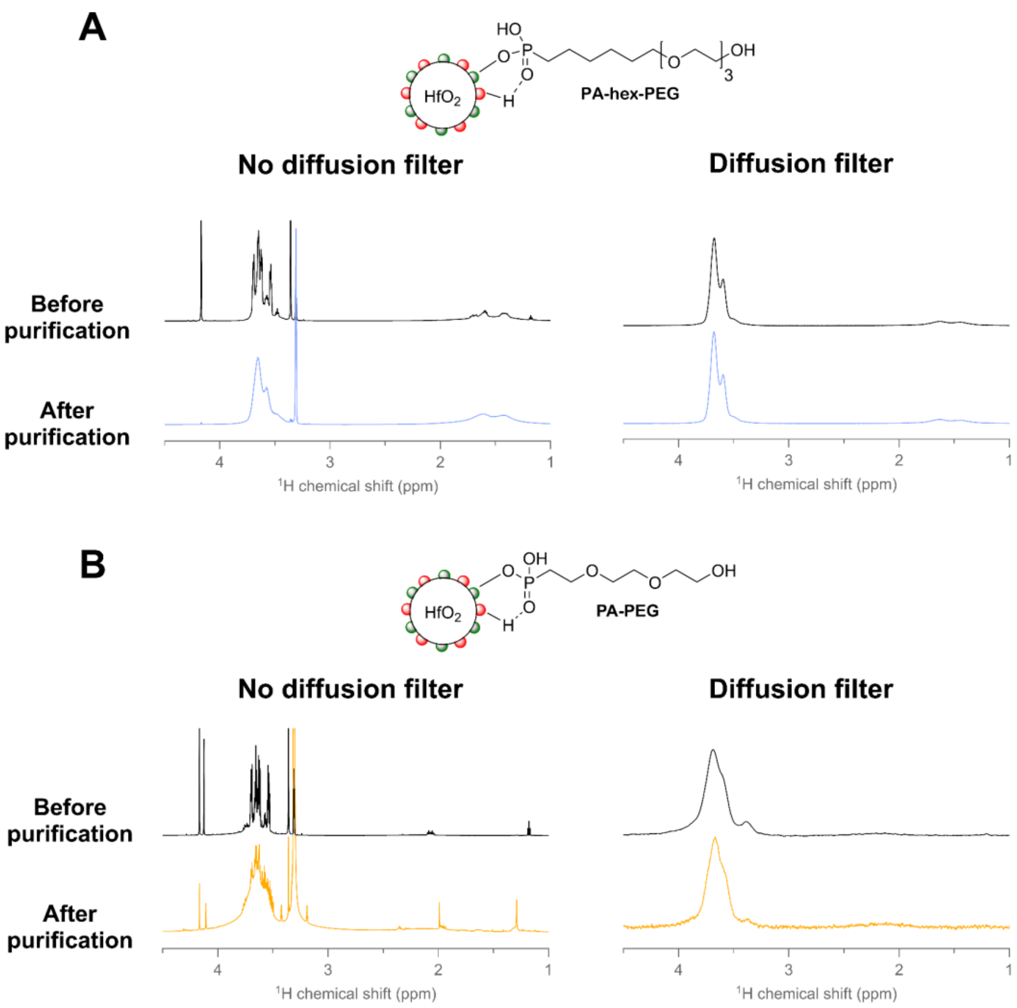

Figure S10: ^{1}H NMR spectra of NCs functionalized with PA-hex-PEG and PA-PEG in MeOD before and after purification using spin filtration. **A:** (Diffusion filtered) ^{1}H NMR spectra of PA-hex-PEG functionalized NCs. **B:** (Diffusion filtered) ^{1}H NMR spectra of PA-PEG functionalized NCs.

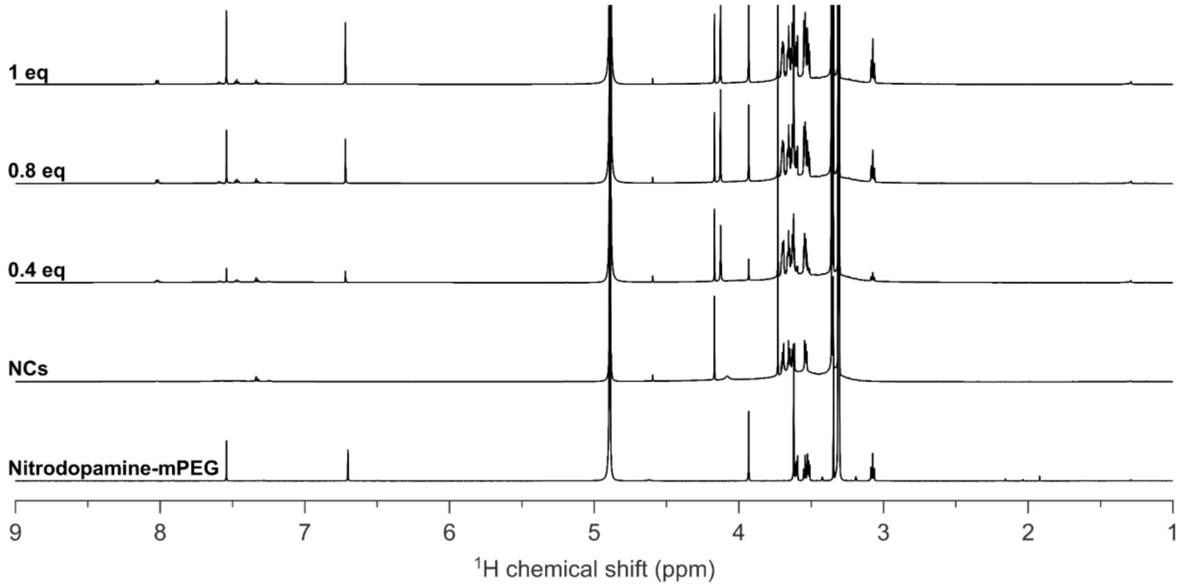

Figure S11: ^{1}H NMR spectra of the attempted ligand exchange between MEEAA functionalized NCs and nitrodopamine-mPEG in MeOD.

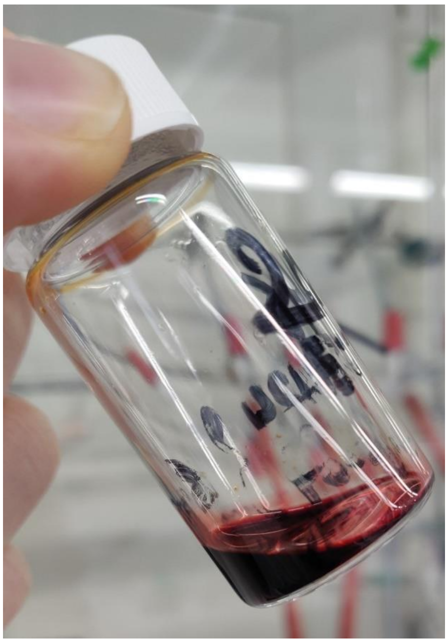

Figure S12: Deprotonation of nitrodopamine-mPEG with NaOD turns the aqueous solution from light yellow to a deep burgundy.

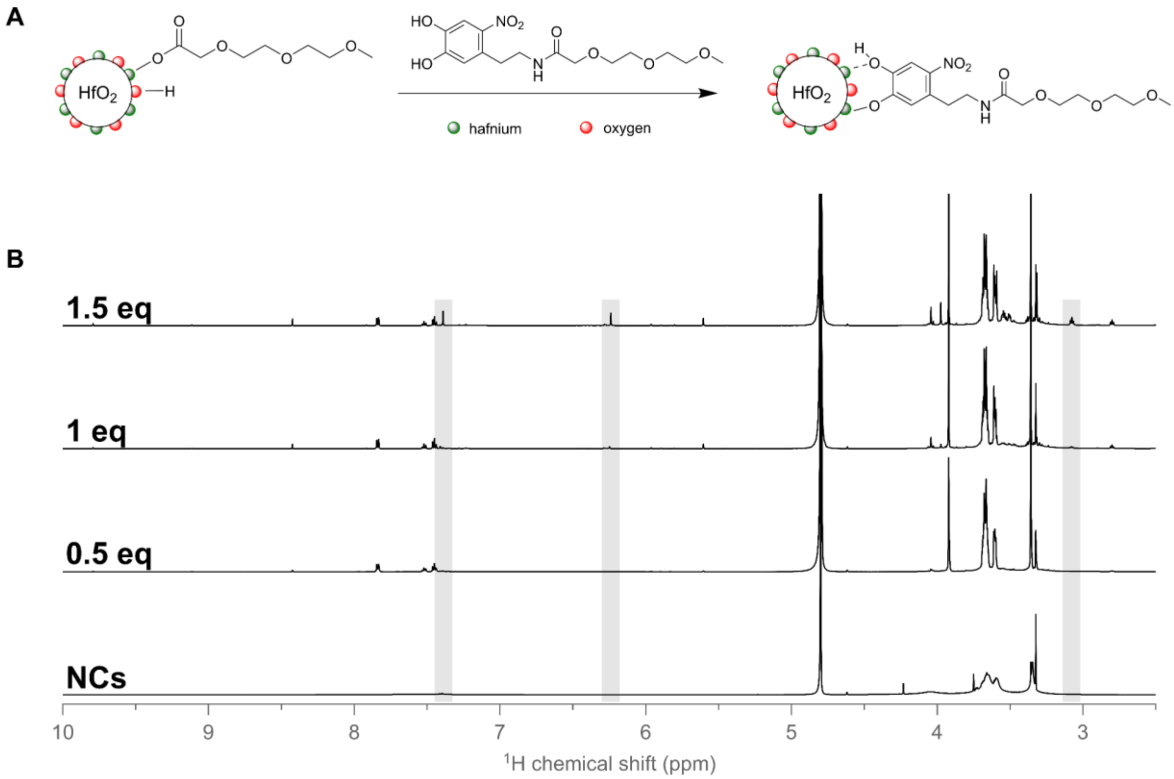

Figure S13: A: Ligand exchange performed between MEEAA functionalized NCs and nitrodopamine-mPEG. **B:** ^1H NMR spectra for the ligand exchange of MEEAA functionalized NCs with nitrodopamine-mPEG in D_2O, performed in steps of 0.5 equivalents. The pH of the solution was kept above 5 during each ligand addition step. Areas of interest are denoted in grey and represent sharp nitrodopamine-mPEG signals in the aromatic region and methylene triplet around 3 ppm.

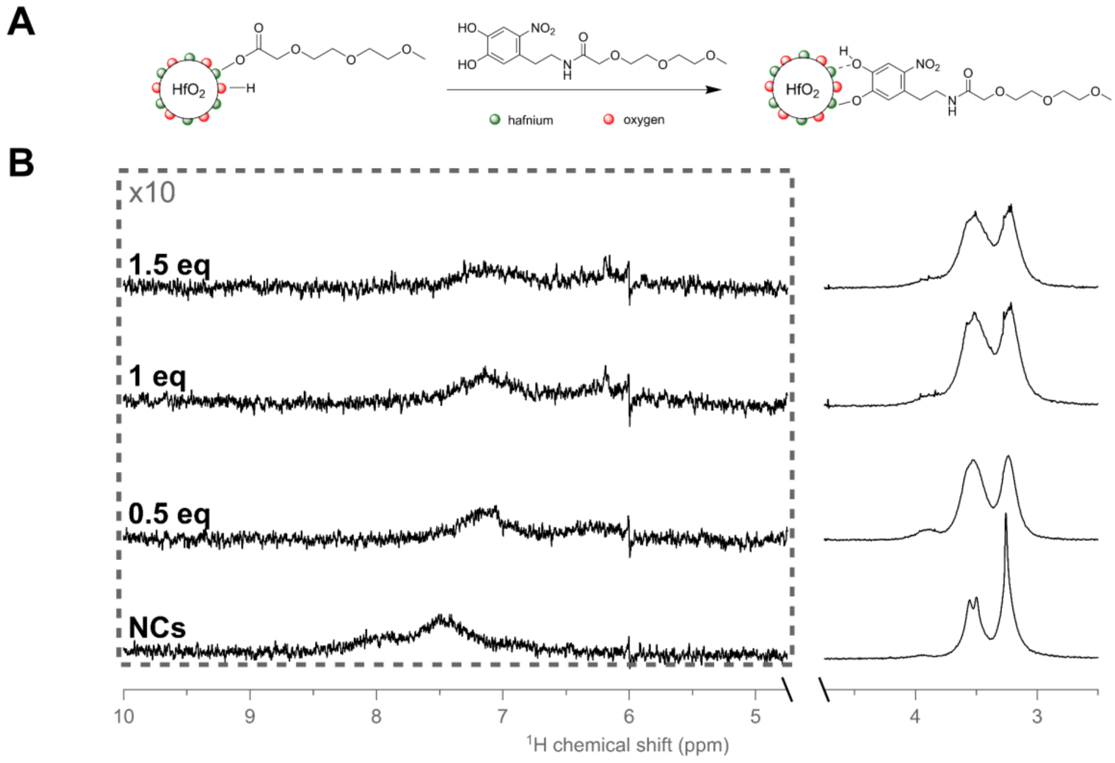

Figure S14: A: Ligand exchange performed between MEEAA functionalized NCs and nitrodopamine-mPEG. **B:** Diffusion filtered ^{1}H NMR spectra for the ligand exchange of MEEAA functionalized NCs with nitrodopamine-mPEG in D_2O, the pH of the solution was kept above 5 during each ligand addition step. The graph has been split in 2 parts each with their own multiplication factor for easier data interpretation.

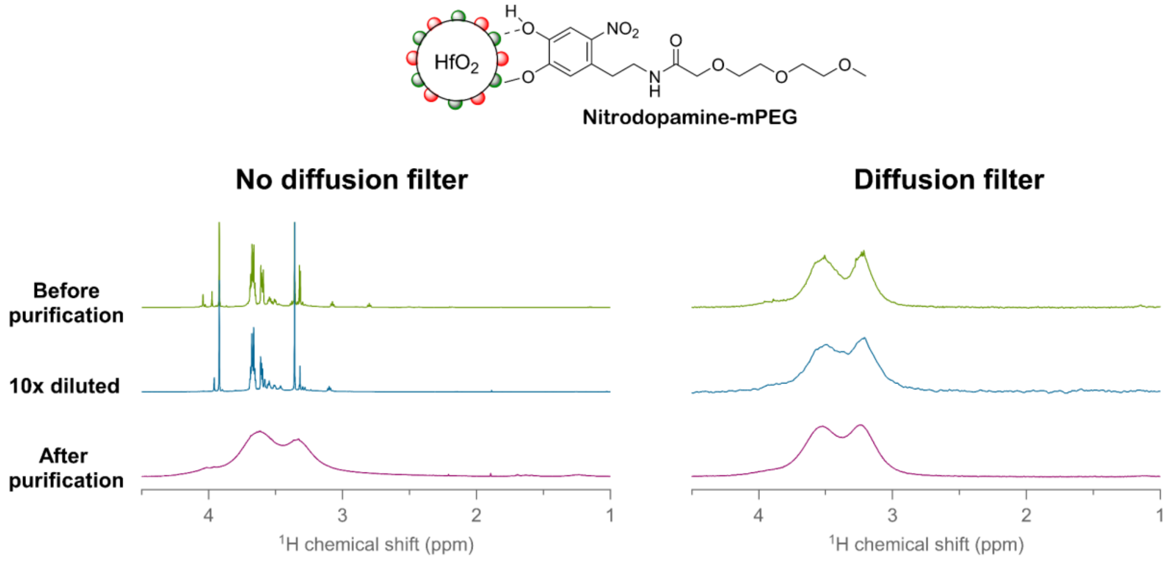

Figure S15: ^{1}H NMR spectra of nitrodopamine-mPEG functionalized NCs in D_2O after addition of 1.5 equivalents of the ligand (top, green), after 10x dilution (middle, blue) with Milli-Q H_2O and after purification (bottom, purple). The left and right side of the figure respectively show ^{1}H spectra and diffusion filtered ^{1}H spectra.

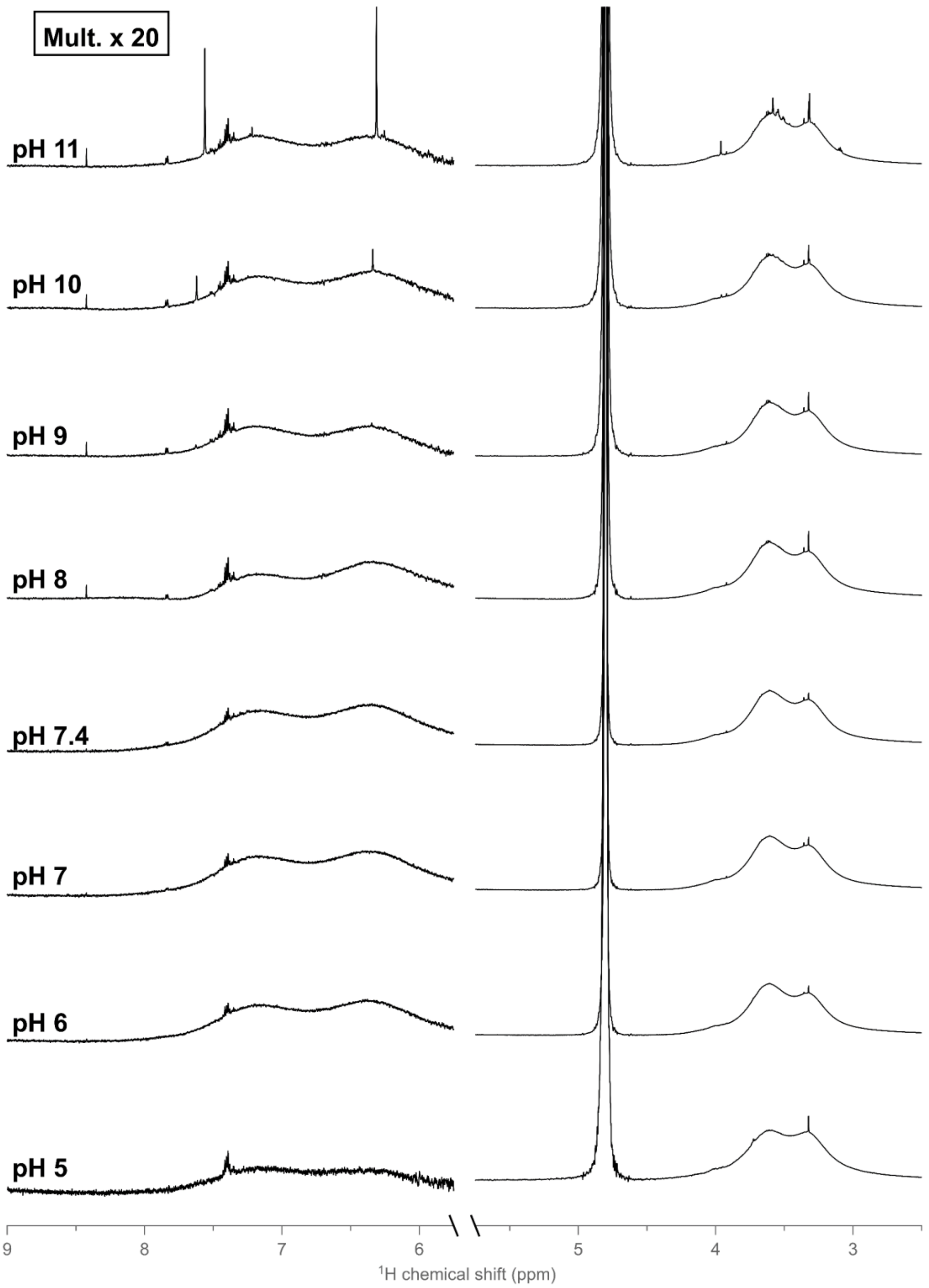

Figure S16: Full range ^{1}H NMR spectra of nitrodopamine-mPEG functionalized NCs in D_2O at different pH values, the graph has been split in 2 parts each with their own multiplication factor for easier data interpretation. Two distinct areas can be distinguished: 6-8 ppm contains aromatic signals arising from nitrodopamine-mPEG binding to the NC surface, the strong broadening is caused by a total loss of rotational freedom of the aromatic protons after binding. 2.5-4.5 ppm contains signals arising from the mPEG ethoxy moieties and two aliphatic CH_2's between the aromatic ring and amide bond.

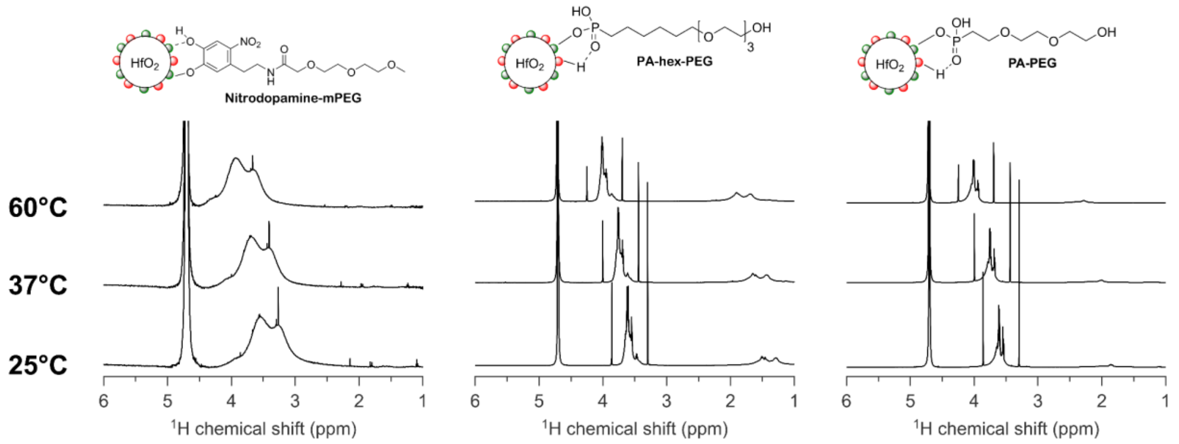

Figure S17: Variable temperature ^{1}H NMR measurements for NCs functionalized with nitrodopamine-mPEG, PA-PEG and PA-hex-PEG at pH 7.4 in D_2O. Ligand binding behavior remains unchanged for all functionalizations, only temperature dependent peak shifting is observed.

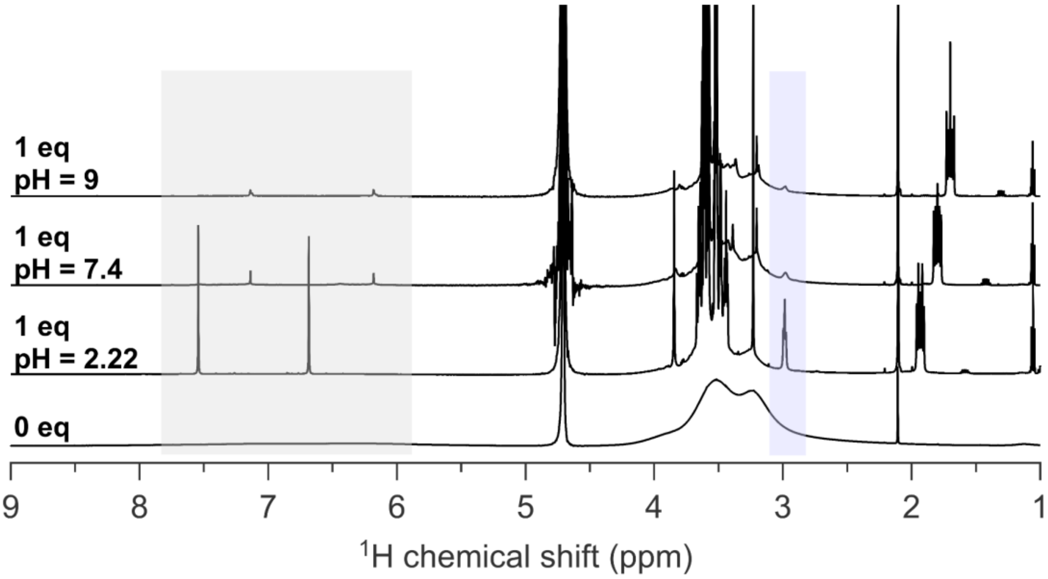

Figure S18: ^{1}H NMR spectra of the competitive ligand exchange between nitrodopamine-mPEG functionalized NCs and PA-PEG at different pH values. Areas of interest are denoted by grey and purple shading.

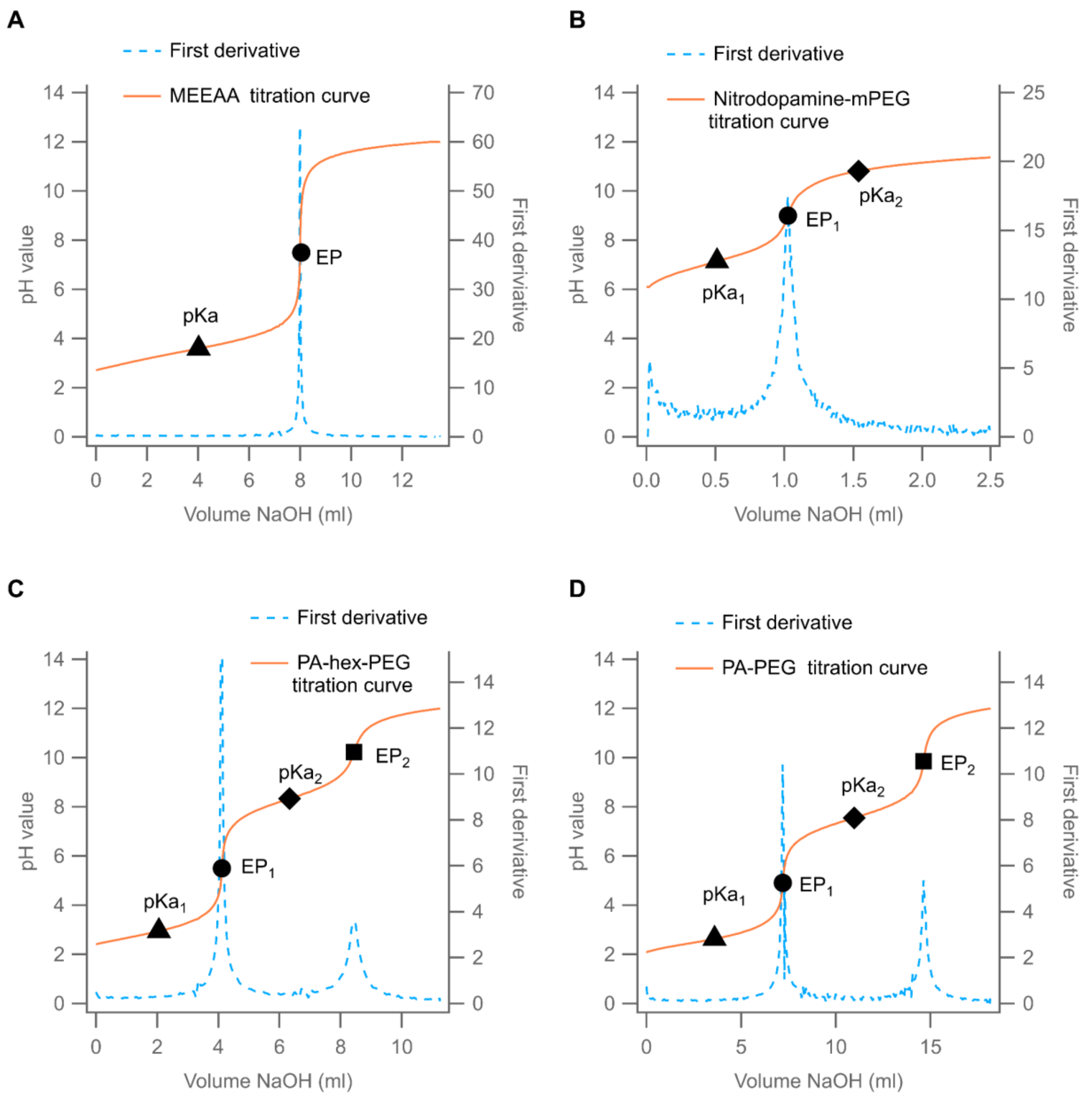

Figure S19: Titration curves for all ligands. All titrations were performed in triplicate and equivalence points were determined by calculating where the first derivatives of the titration curve are maximized. **A**: Titration curve for MEEAA, **B**: titration curve for nitrodopamine-mPEG, **C**: titration curve for PA-hex-PEG, **D**: titration curve for PA-PEG.

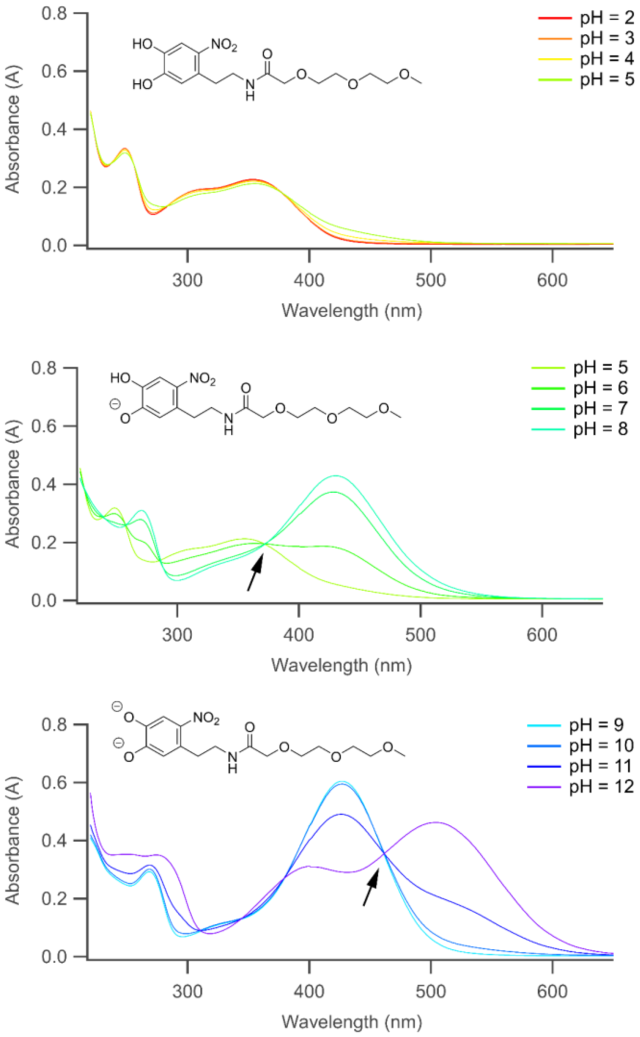

Figure S20: UV-Vis spectra of freely diffusing nitrodopamine-mPEG at different pH values in H_2O. Ligand deprotonation states are shown at different pH values and isobestic points are denoted by arrows.

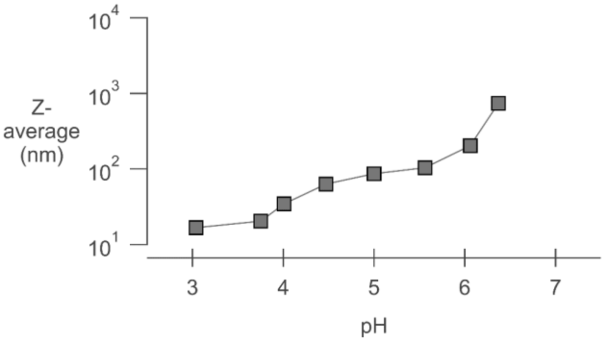

Figure S21: Effect of pH on colloidal stability in water for purified NCs functionalized with MEEAA.

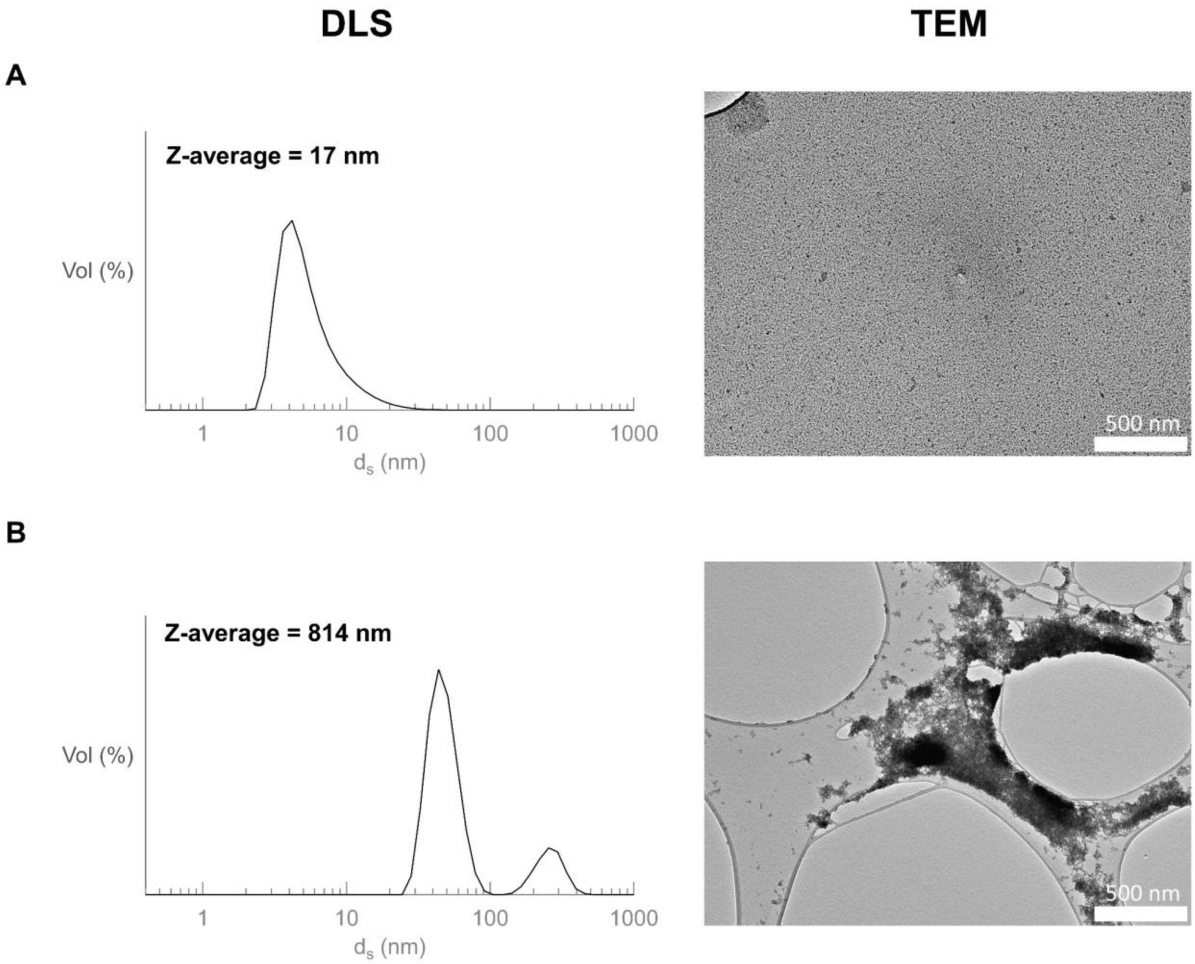

Figure S22: A side-by-side comparison of DLS (Z-average values and the size distribution) with TEM images of the same sample. **A:** MEEAA functionalized NCs in water at pH 3**. B:** MEEAA functionalized NCs in water at pH 6.8.

3. Spectra of synthesized compounds

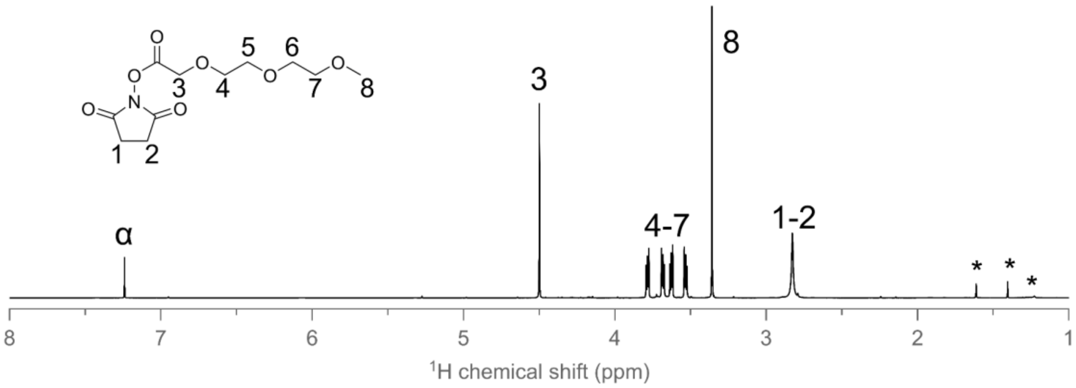

Figure S23: ^{1}H NMR spectrum of MEEAA-NHS in $CDCl_3$.

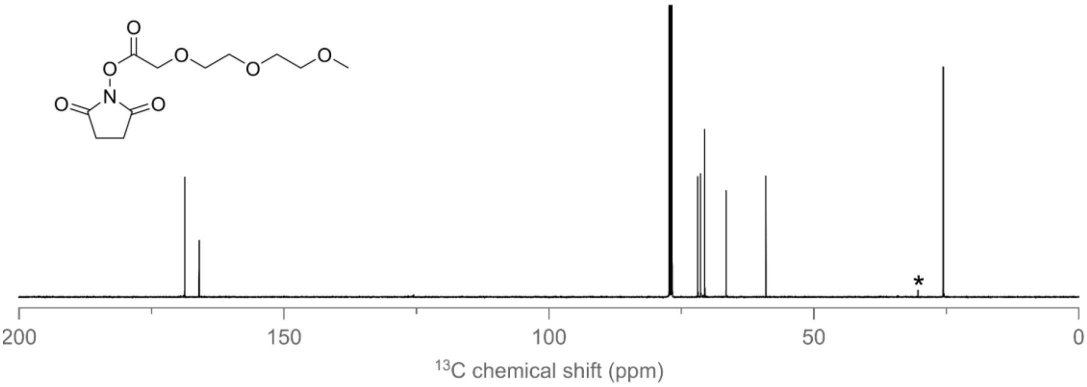

Figure S24: Proton decoupled ^{13}C NMR spectrum of MEEAA-NHS in $CDCl_3$.

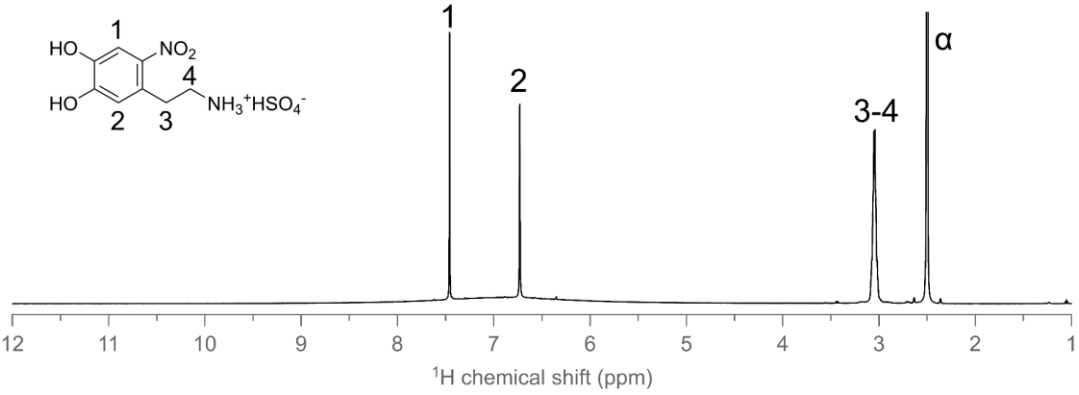

Figure S25: ^{1}H NMR spectrum of nitrodopamine hemisulfate in DMSO-d_6.

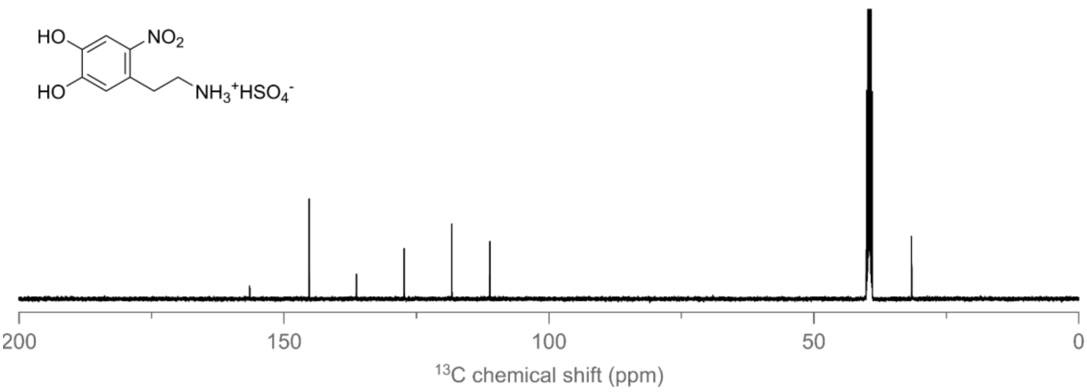

Figure S26: Proton decoupled ^{13}C NMR spectrum of nitrodopamine hemisulfate in DMSO-d_6.

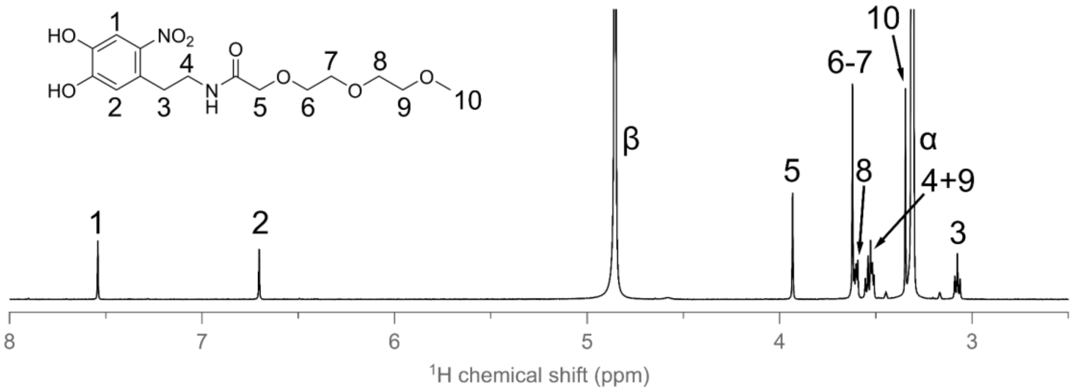

Figure S27: ^{1}H NMR spectrum of nitrodopamine-mPEG in MeOD.

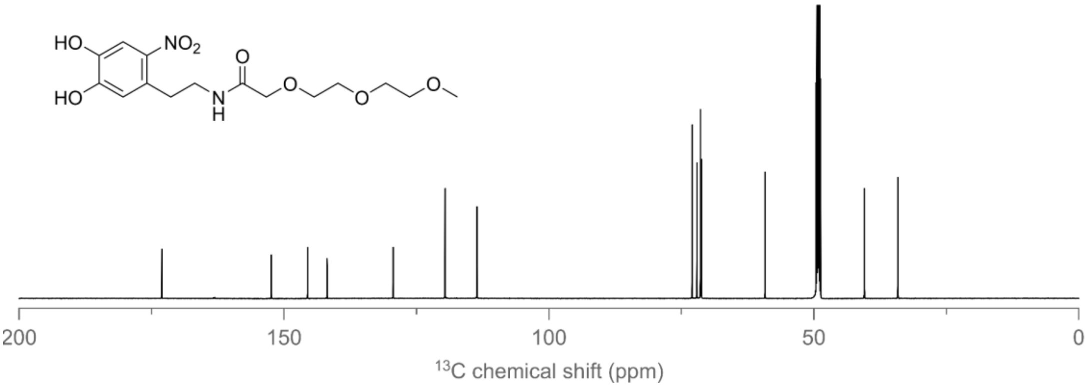

Figure S28: Proton decoupled ^{13}C NMR spectrum of nitrodopamine-mPEG in MeOD.

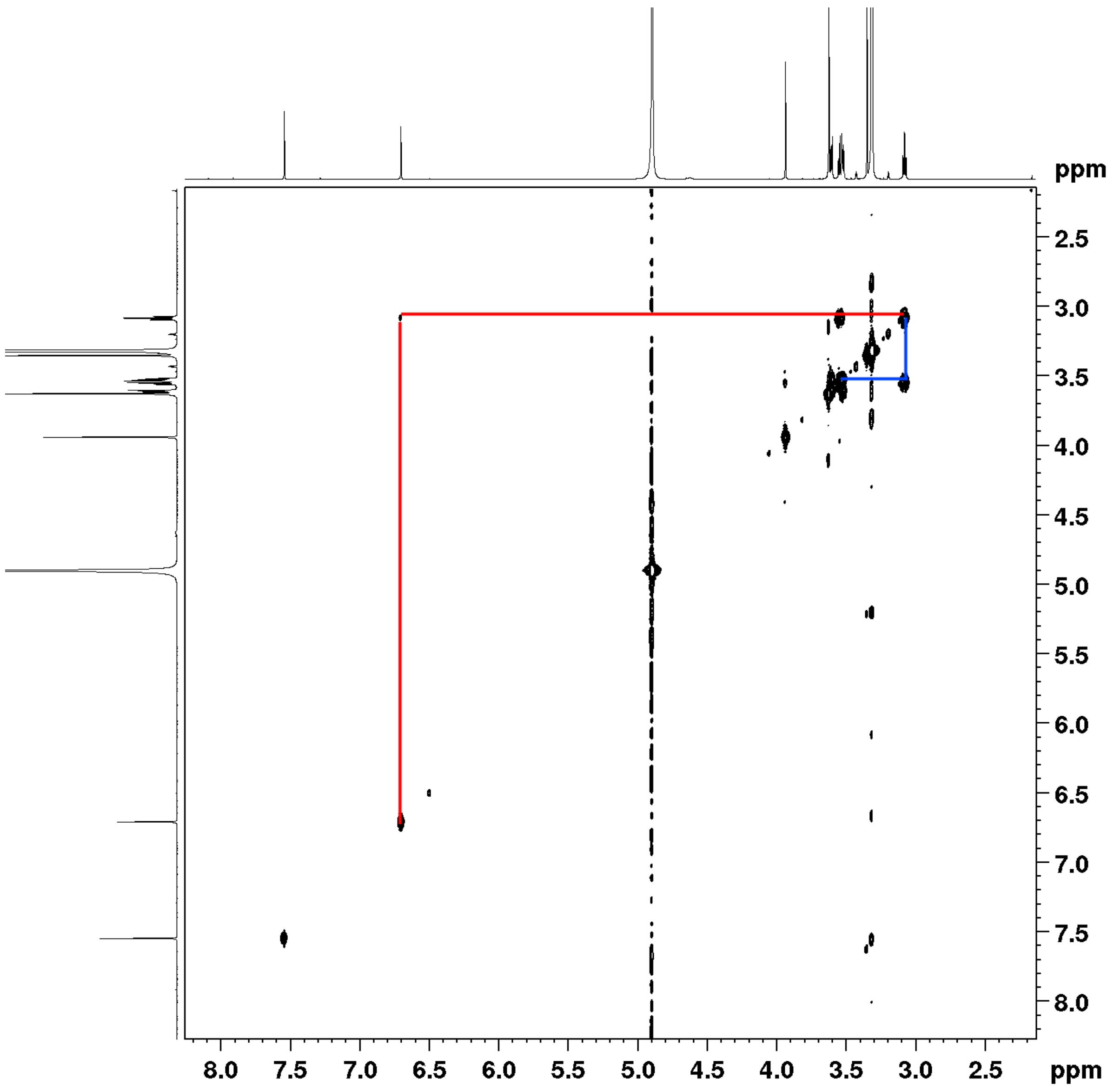

Figure S29: Full range 2D COSY NMR spectrum of nitrodopamine-mPEG in MeOD.

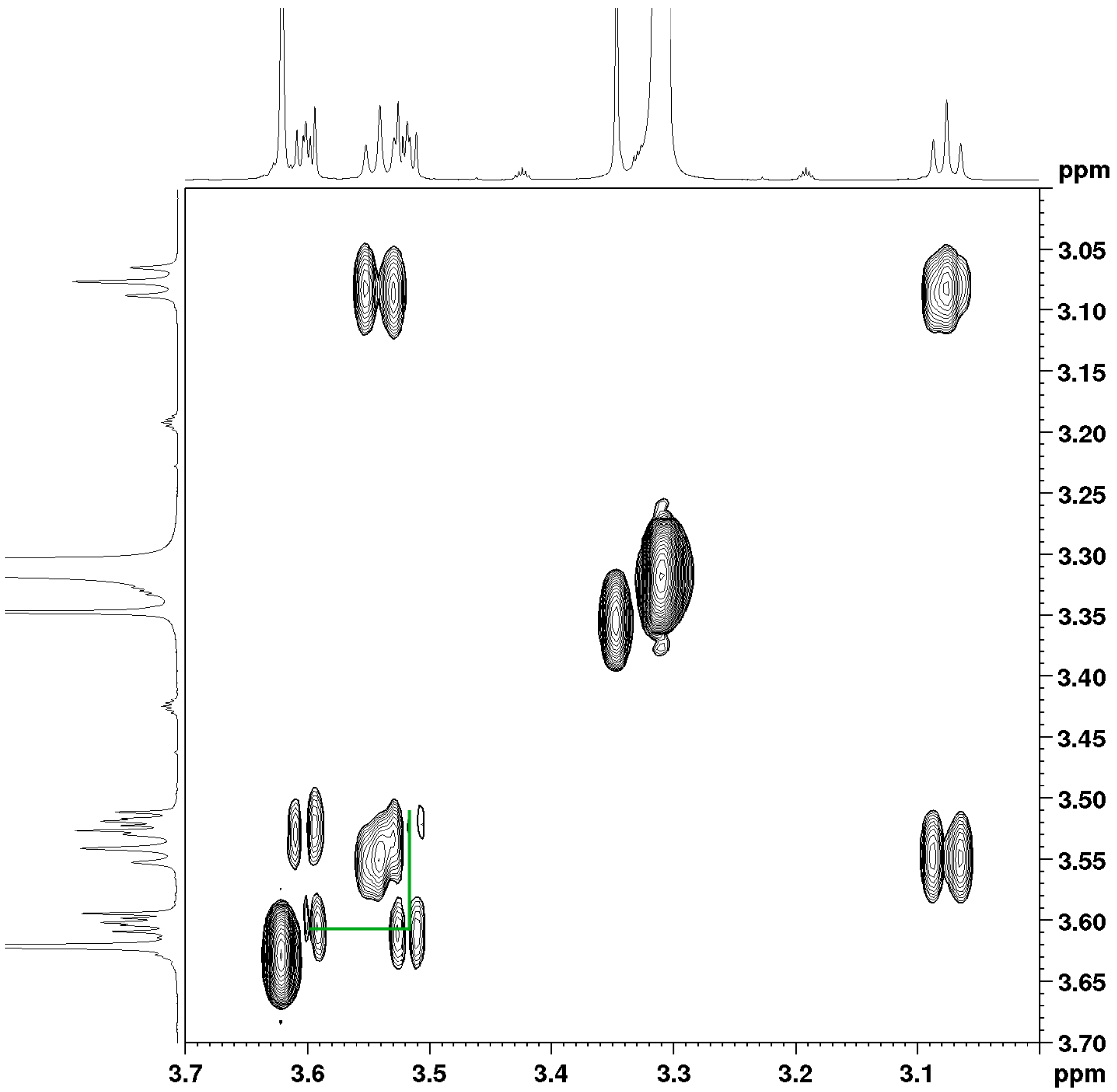

Figure S30: 2D COSY NMR spectrum focussed between 3-4 ppm of nitrodopamine-mPEG in MeOD.

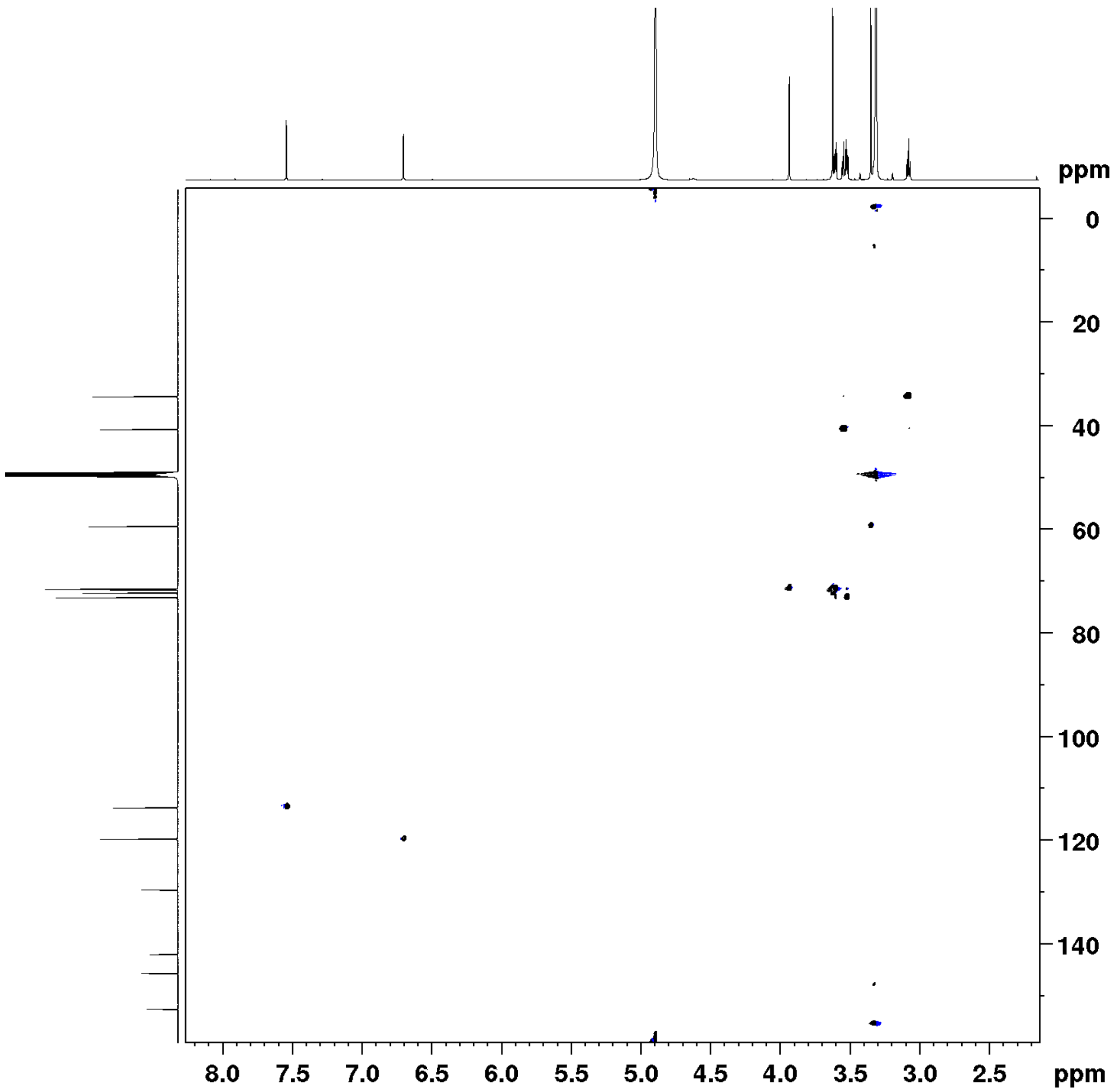

Figure S31: 2D HSQC NMR spectrum of nitrodopamine-mPEG in MeOD.

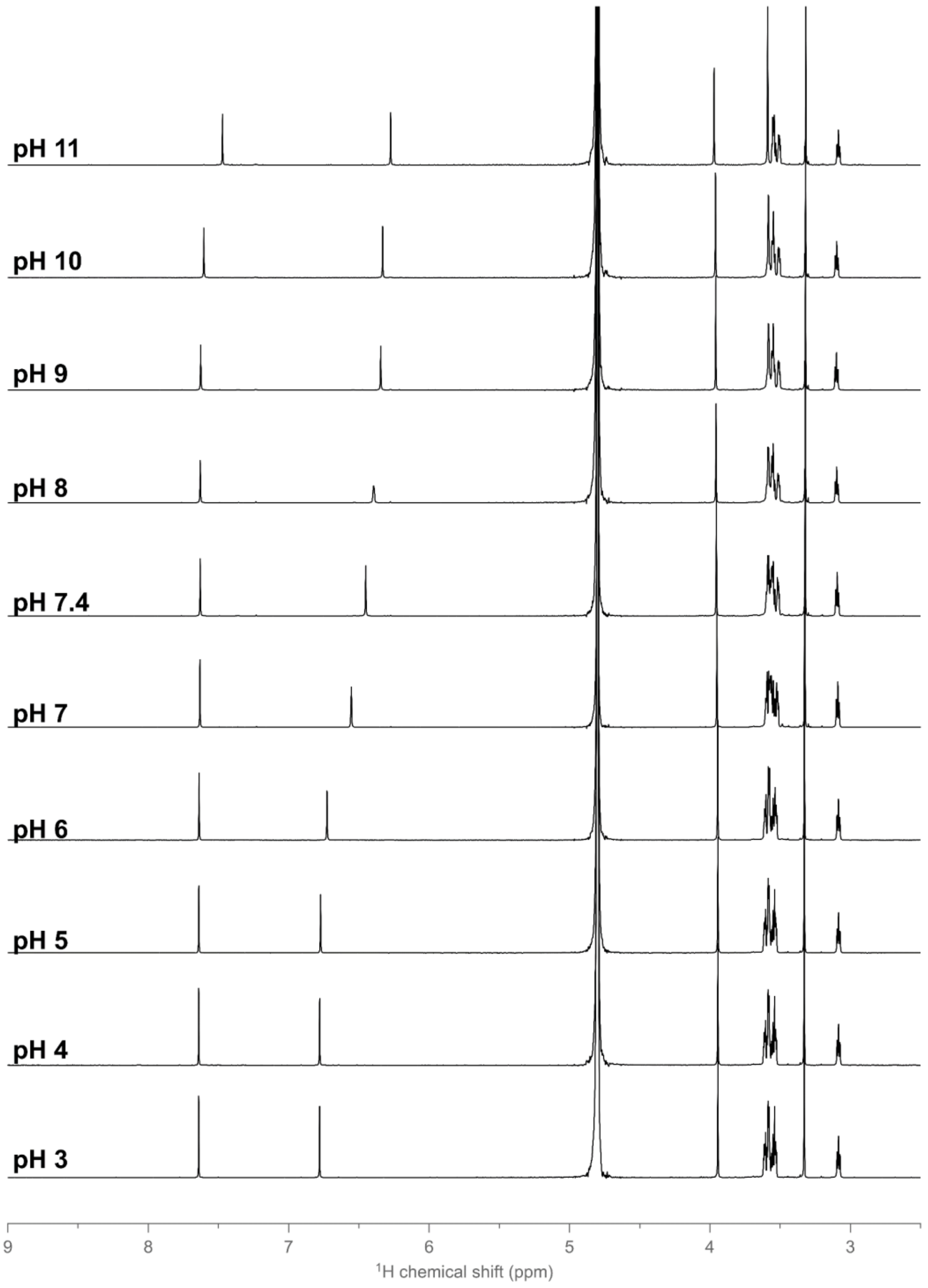

Figure S32: Reference ^{1}H spectra of nitrodopamine-mPEG in D_2O at different pH values. A clear change of chemical environment can be observed for the aromatic peaks as the pH changes, caused by deprotonation of the catechol hydroxyl functional groups.

4. ^{31}P peak deconvolution - D_2O titration

D_2O percentage	Peak fit	Peak areas
0%	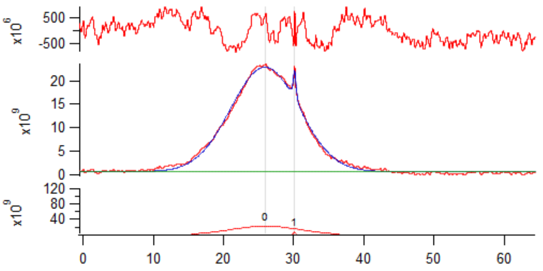	**Total Peak Area** = 2.767e+11 +/- 1.0673e+08<br><br>**Peak 0 Type: Gauss (bound peak)**<br>Area = 2.72e+11 +/- 1.00e+08<br><br>**Peak 1 Type: Lorentzian (unbound peak)**<br>Area = 4.42e+09 +/- 3.64e+07<br><br>**Free ligand fraction** = 0.016
25%	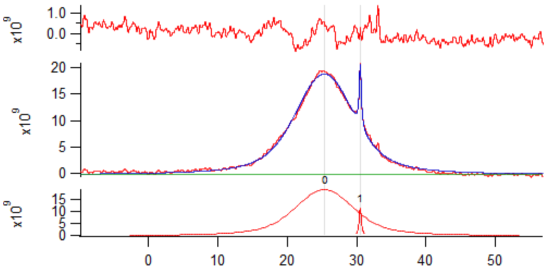	**Total Peak Area** = 2.5462e+11 +/- 1.18e+08<br><br>**Peak 0 Type: Voigt (bound peak)**<br>Area = 2.48e+11 +/-1.17e+08<br><br>**Peak 1 Type: Lorentzian (unbound peak)**<br>Area = 6.70e+09 +/- 1.39e+07<br><br>**Free ligand fraction** = 0.026
50%	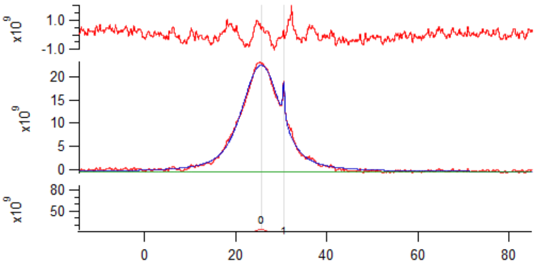	**Total Peak Area** = 3.0757e+11 +/- 1.5005e+08<br><br>**Peak 0 Type: Voigt (bound peak)**<br>Area = 3.01e+11+/- 1.47e+08<br><br>**Peak 1 Type: Lorentzian (unbound peak)**<br>Area = 6.18e+09 +/- 3.10e+07<br><br>**Free ligand fraction** = 0.020
75%	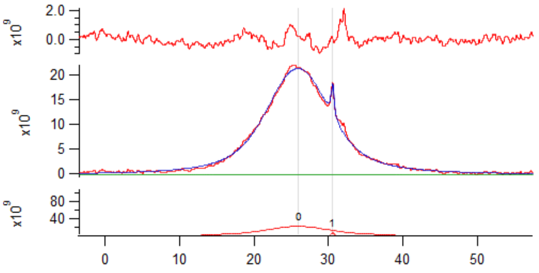	**Total Peak Area** = 2.9914e+11 +/- 1.734e+08<br><br>**Peak 0 Type: Voigt (bound peak)**<br>Area = 2.94e+11 +/- 1.70e+08<br><br>**Peak 1 Type: Lorentzian (unbound peak)**<br>Area = 5.05e+09 +/- 3.26e+07<br><br>**Free ligand fraction** = 0.017
100%		**Total Peak Area** = 2.8705e+11 +/- 1.7539e+08

	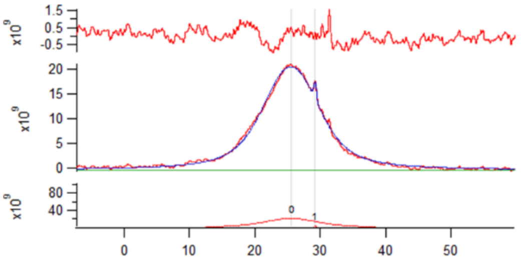	**Peak 0 Type: Voigt (bound peak)**<br>Area = 2.85e+11 +/- 1.75e+08<br><br>**Peak 1 Type: Gauss (unbound peak)**<br>Area = 1.84e+09 +/- 1.53e+07<br><br>**Free ligand fraction** = 0.0064

Table 1: ^{31}P spectra of PA-hex-PEG functionalized NCs in MeOD at different points during the titration with D_2O. Peak deconvolution was performed on the spectrally overlapping bound and unbound ligand peaks. Peak fit type was chosen based on achieving the lowest amount of residuals.

D_2O percentage	Peak fit	Peak areas
0%	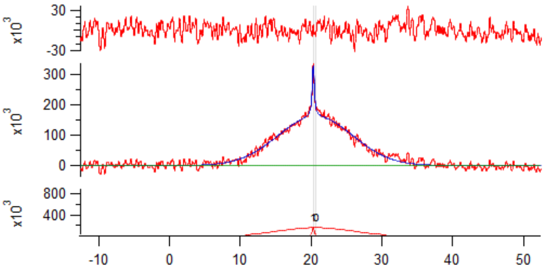	**Total Peak Area** = 2.2379e+06 +/- 2355.8<br><br>**Peak 0 Type: Gauss (bound peak)**<br>Area = 2.14e+06 +/- 2283.7<br><br>**Peak 1 Type: Lorentzian (unbound peak)**<br>Area = 94848 +/- 578.32<br><br>**Free ligand fraction** = 0.042
25%	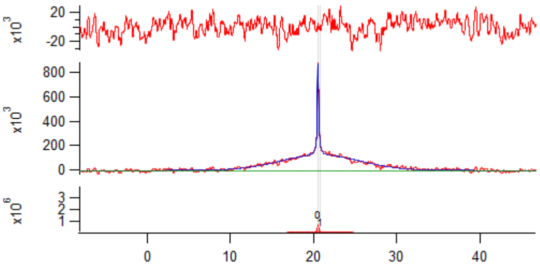	**Total Peak Area** = 1.7516e+06 +/- 2408.3<br><br>**Peak 0 Type: Lorentzian (unbound peak)**<br>Area = 2.36e+05 +/- 423.98<br><br>**Peak 1 Type: Gauss (bound peak)**<br>Area = 1.52e+06 +/- 2370.7<br><br>**Free ligand fraction** = 0.135
50%	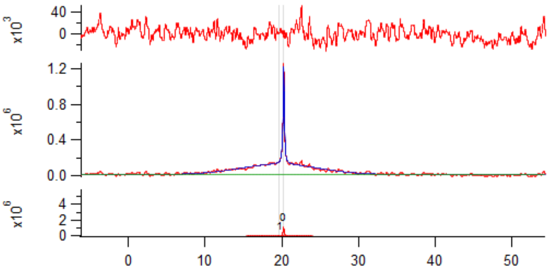	**Total Peak Area** = 1.8115e+06 +/- 2725.5<br><br>**Peak 0 Type: Lorentzian (unbound peak)**<br>Area = 3.31e+05 +/- 454.24<br><br>**Peak 1 Type: Gauss (bound peak)**<br>Area = 1.48e+06 +/- 2687.4<br><br>**Free ligand fraction** = 0.183
75%		**Total Peak Area** = 1.8812e+06 +/- 2111.4

	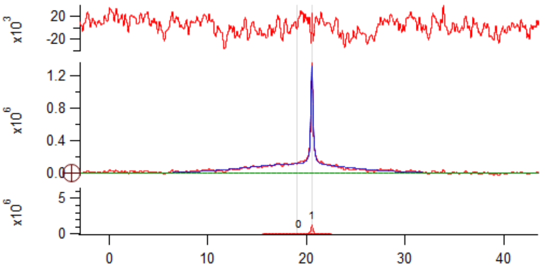	**Peak 0 Type: Gauss (bound peak)**<br>Area = 1.45e+06 +/- 2058.1<br><br>**Peak 1 Type: Lorentzian (unbound peak)**<br>Area = 4.29e+05 +/- 471.2<br><br>**Free ligand fraction** = 0.228
100%	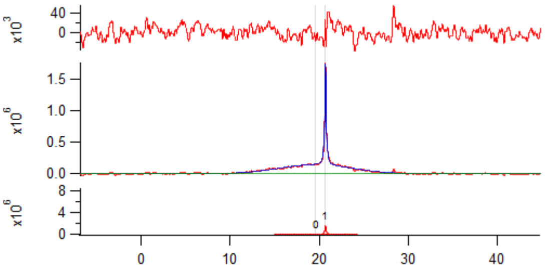	**Total Peak Area** = 1.9663e+06 +/- 2404.4<br><br>**Peak 0 Type: Gauss (bound peak)**<br>Area = 1.50e+06 +/-2360.7<br><br>**Peak 1 Type: Lorentzian (unbound peak)**<br>Area = 4.71e+05 +/- 456.11<br><br>**Free ligand fraction** = 0.239

Table 2: ^{31}P spectra of PA-PEG functionalized NCs in MeOD at different points during the titration with D_2O. Peak deconvolution was performed on the spectrally overlapping bound and unbound ligand peaks. Peak fit type was chosen based on achieving the lowest amount of residuals.

5. ^{31}P and ^{1}H peak deconvolution - pH titration

pH value	Peak fit	Peak areas
10	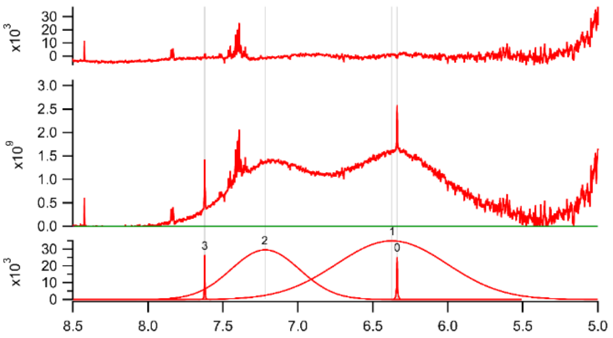	**Total Peak Area** = 49569 +/- 299.29<br><br>**Peak 0 Type: Lorentzian (unbound peak)**<br>Area = 219.36 +/- 15.684<br><br>**Peak 1 Type: Gauss (bound peak)**<br>Area = 32265 +/- 236.69<br><br>**Peak 2 Type: Gauss (bound peak)**<br>Area = 16937 +/- 182.08<br><br>**Peak 3 Type: Lorentzian (unbound peak)**<br>Area = 147.71 +/- 12.312<br><br>**Free ligand fraction** = 0.0074
11		**Total Peak Area** = 48350 +/- 140.95

	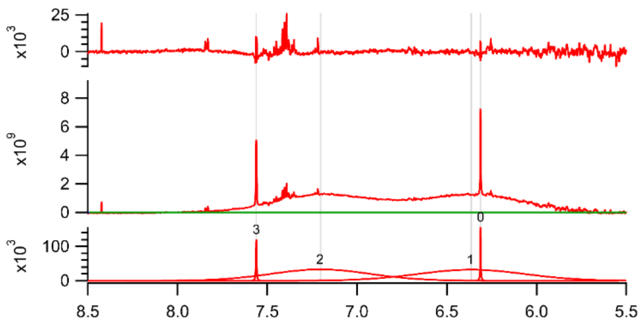	**Peak 0 Type: Lorentzian (unbound peak)**<br>Area = 805.75 +/- 5.4806<br><br>**Peak 1 Type: Gauss (bound peak)**<br>Area = 24013 +/- 100.7<br><br>**Peak 2 Type: Gauss (bound peak)**<br>Area = 22746 +/- 98.281<br><br>**Peak 3 Type: Lorentzian (unbound peak)**<br>Area = 785.19 +/- 6.2167<br><br>**Free ligand fraction** = 0.033

Table 3: ^{1}H spectra of nitrodopamine-mPEG functionalized NCs in D_2O at different pH values. Peak deconvolution was performed on the spectrally overlapping bound and unbound ligand peaks. Peak fit type was chosen based on achieving the lowest amount of residuals.

pH value	Peak fit	Peak areas
3.39	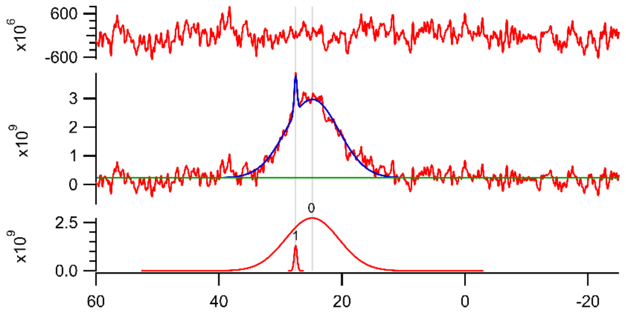	**Total Peak Area =** 4.336e+10 +/- 1.0985e+08<br><br>**Peak 0 Type: Lorentzian (bound peak)**<br>Area = 4.1557e+10 +/- 1.0543e+08<br><br>**Peak 1 Type: Lorentzian (unbound peak)**<br>Area = 1.8036e+09 +/- 3.0851e+07<br><br>**Free ligand fraction** = 0.042
6	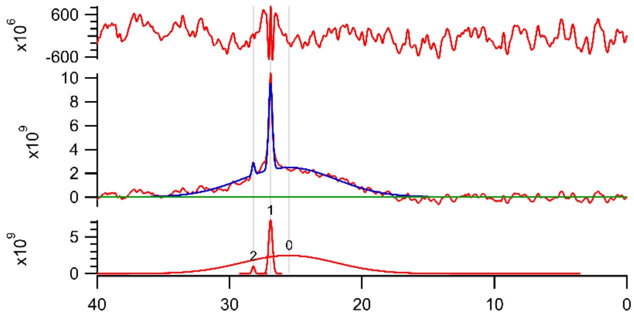	**Total Peak Area =** 2.492e+10 +/- 5.7931e+07<br><br>**Peak 0 Type: Gauss (bound peak)**<br>Area = 2.0869e+10 +/- 5.4624e+07<br><br>**Peak 1 Type: Lorentzian (unbound peak)**<br>Area = 3.6902e+09 +/- 1.649e+07<br>**Peak 2 Type: Lorentzian (unbound impurity)**

		Area = 3.5928e+08 +/- 1.0013e+07<br><br>**Free ligand fraction** = 0.15
8	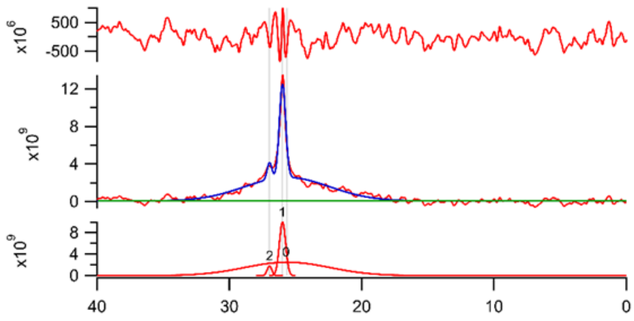	**Total Peak Area** = 2.802e+10 +/- 5.6498e+07<br><br>**Peak 0 Type: Gauss (bound peak)**<br>Area = 1.8554e+10 +/- 5.5402e+07<br><br>**Peak 1 Type: Lorentzian (unbound peak)**<br>Area = 8.7166e+09 +/- 6.7308e+06<br><br>**Peak 2 Type: Lorentzian (unbound impurity)**<br>Area = 7.4916e+08 +/- 8.79e+06<br><br>**Free ligand fraction** = 0.32
9.5	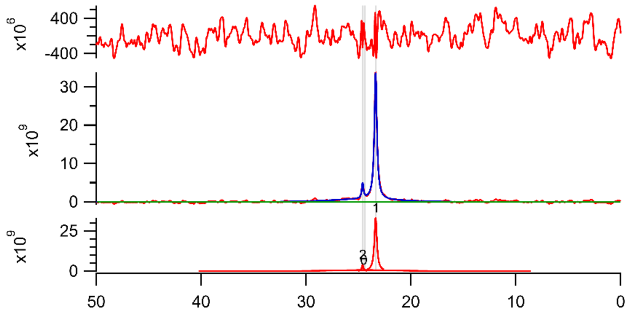	**Total Peak Area** = 2.148e+10 +/- 4.7858e+07<br><br>**Peak 0 Type: Gauss (bound peak)**<br>Area = 5.2345e+09 +/- 4.3987e+07<br><br>**Peak 1 Type: Lorentzian (unbound peak)**<br>Area = 1.5087e+10 +/- 1.5255e+07<br><br>**Peak 2 Type: Lorentzian (unbound impurity)**<br>Area = 1.1586e+09 +/- 1.108e+07<br><br>**Free ligand fraction** = 0.74

Table 4: ^{31}P spectra of PA-hex-PEG functionalized NCs in D_2O at different pH values. Peak deconvolution was performed on the spectrally overlapping bound and unbound ligand peaks. Peak fit type was chosen based on achieving the lowest amount of residuals.

pH value	Peak fit	Peak areas
3.3	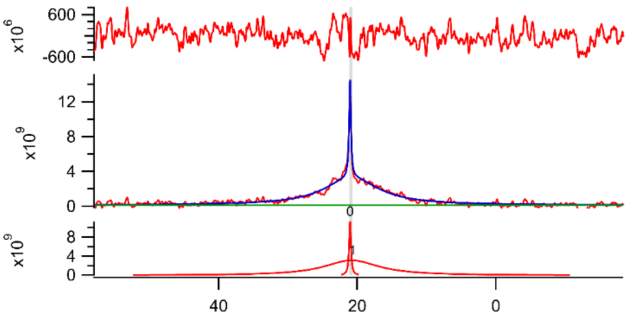	**Total Peak Area** = 5.312e+10 +/- 1.0899e+08<br><br>**Peak 0 Type: Lorentzian (unbound peak)**<br>Area =5.5325e+09 +/- 1.4676e+07<br><br>**Peak 1 Type: Lorentzian (bound peak)**<br>Area = 4.7591e+10 +/- 1.0799e+08<br><br>**Free ligand fraction** = 0.104
5.9	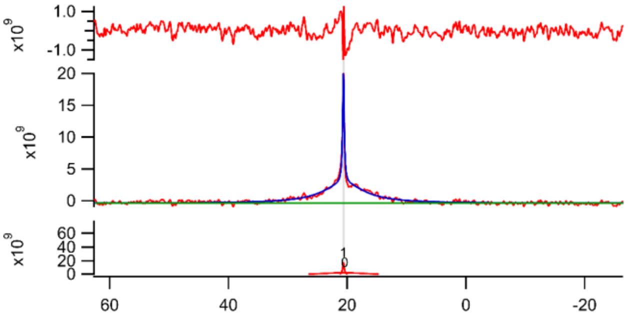	**Total Peak Area** = 5.046e+10 +/- 7.2167e+07<br><br>**Peak 0 Type: Lorentzian (bound peak)**<br>Area = 4.1682e+10 +/- 7.1525e+07<br><br>**Peak 1 Type: Lorentzian (unbound peak)**<br>Area = 8.7738e+09 +/- 9.6049e+06<br><br>**Free ligand fraction** = 0.174
6.9	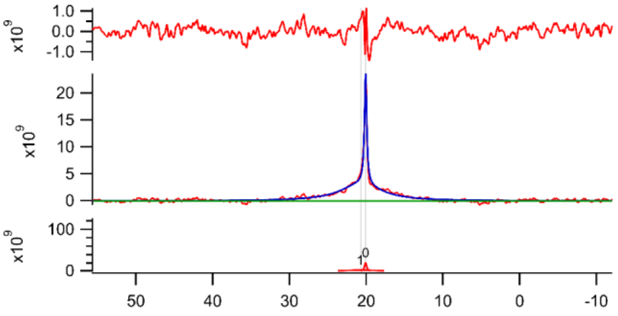	**Total Peak Area** = 4.366e+10 +/- 7.044e+07<br><br>**Peak 0 Type: Lorentzian (unbound peak)**<br>Area = 1.141e+10 +/- 6.5095e+06<br><br>**Peak 1 Type: Lorentzian (bound peak)**<br>Area = 3.2252e+10 +/- 7.0139e+07<br><br>**Free ligand fraction** = 0.26
7.82		**Total Peak Area** = 3.873e+10 +/- 4.7e+07<br><br>**Peak 0 Type: Lorentzian (unbound peak)**

	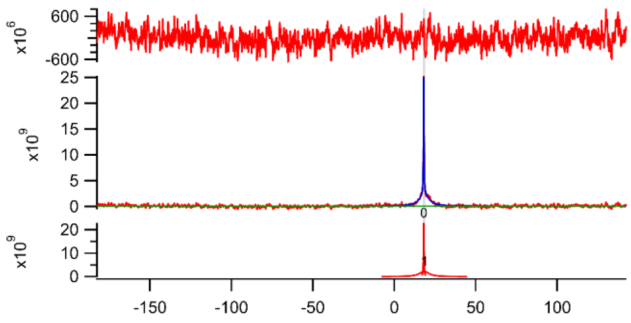	Area = 1.3866e+10 +/- 5.358e+06<br><br>**Peak 1 Type: Lorentzian (bound peak)**<br>Area = 2.4866e+10 +/- 4.6693e+07<br><br>**Free ligand fraction** = 0.36
8.73	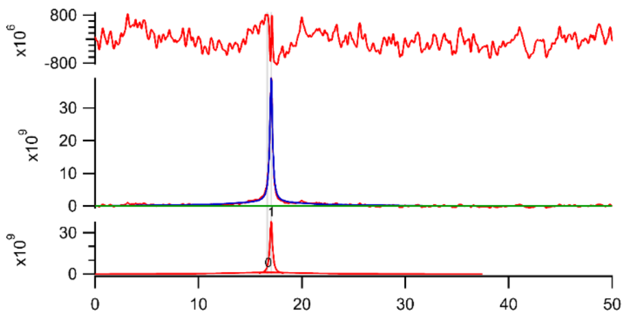	**Total Peak Area** = 3.5457e+10 +/- 6.1775e+07<br><br>**Peak 0 Type: Lorentzian (bound peak)**<br>Area = 1.6866e+10 +/- 5.954e+07<br><br>**Peak 1 Type: Lorentzian (unbound peak)**<br>Area = 1.8591e+10 +/- 1.6466e+07<br><br>**Free ligand fraction** = 0.53
9.6	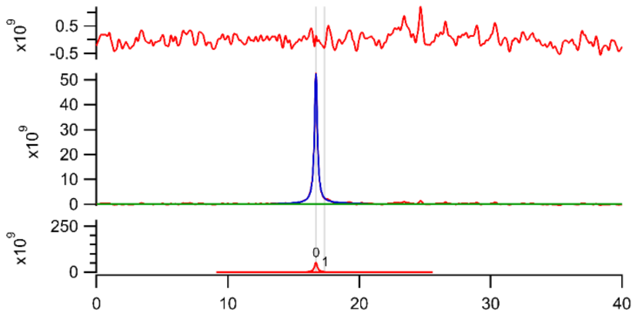	**Total Peak Area** = 2.259e+10 +/- 3.0297e+07<br><br>**Peak 0 Type: Lorentzian (unbound peak)**<br>Area = 2.1221e+10 +/- 1.2576e+07<br><br>**Peak 1 Type: Gauss (bound peak)**<br>Area = 1.3715e+09 +/- 2.7564e+07<br><br>**Free ligand fraction** = 0.94

Table 5: ^{31}P spectra of PA-PEG functionalized NCs in D_2O at different pH values. Peak deconvolution was performed on the spectrally overlapping bound and unbound ligand peaks. Peak fit type was chosen based on achieving the lowest amount of residuals.

References

1. Ashiotis, G.; Deschildre, A.; Nawaz, Z.; Wright, J. P.; Karkoulis, D.; Picca, F. E.; Kieffer, J., The fast azimuthal integration Python library: pyFAI. *Journal of Applied Crystallography* **2015,** *48* (2), 510-519.
2. Wright, C. J.; Zhou, X.-D., Computer-assisted area detector masking. *Journal of Synchrotron Radiation* **2017,** *24* (2), 506-508.
3. Juhas, P.; Davis, T.; Farrow, C. L.; Billinge, S. J. L., PDFgetX3: a rapid and highly automatable program for processing powder diffraction data into total scattering pair distribution functions. *Journal of Applied Crystallography* **2013,** *46* (2), 560-566.
4. Yang, X.; Juhás, P.; Farrow, C. L.; Billinge, S. J. L., xPDFsuite: an end-to-end software solution for high throughput pair distribution function transformation, visualization and analysis. *arXiv: Materials Science* **2014**.
5. Juhas, P.; Farrow, C. L.; Yang, X.; Knox, K. R.; Billinge, S. J. L., Complex modeling: a strategy and software program for combining multiple information sources to solve ill posed structure and nanostructure inverse problems. *Acta Crystallographica Section A* **2015,** *71* (6), 562-568.
6. Akoka, S.; Barantin, L.; Trierweiler, M., Concentration Measurement by Proton NMR Using the ERETIC Method. *Analytical Chemistry* **1999,** *71* (13), 2554-2557.
